# Supplementary material for: Rationally designed Campylobacter jejuni Cas9 enables efficient gene activation and base editing
Source: Mol Ther Nucleic Acids. 2024 Oct 18;35(4):102366. doi: 10.1016/j.omtn.2024.102366 (PMC11570936; doi:10.1016/j.omtn.2024.102366)
Supplement: Document S1. Figures S1–S10 and Tables S1–S7 [file mmc1.pdf]

OMTN, Volume *35*

Supplemental information

Rationally designed *Campylobacter jejuni* Cas9 enables efficient gene activation and base editing

Yuxi Chen, Rui Kang, Yuanling Jiang, Qi Zheng, Yue Yang, Jiaqi Liu, Guanglan Wu, Weijun Zhao, Zhan Li, Chengxiang Peng, Pengfei Zhang, Fei Peng, Qianyi Liu, Sihui Hu, Xiao Luo, Guifang Wu, Kaixin Cui, Junjiu Huang, Yongming Wang, Zhou Songyang, and Puping Liang

Figure S1

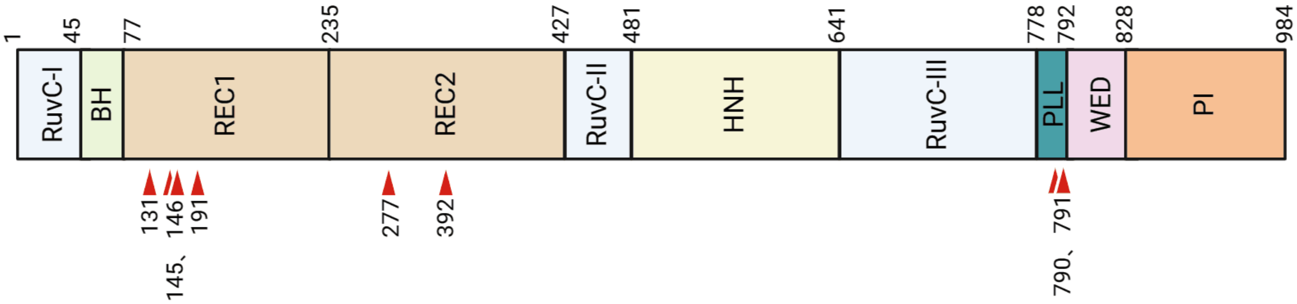

Figure S1: Schematic Overview of Mutations in CjCas9. This schematic provides a detailed view of the mutations introduced in the CjCas9 protein. The numbers correspond to the specific amino acid positions within the protein's structural domains. The red triangles denote the clarity locations where point mutations have been introduced within these domains.

Figure S2

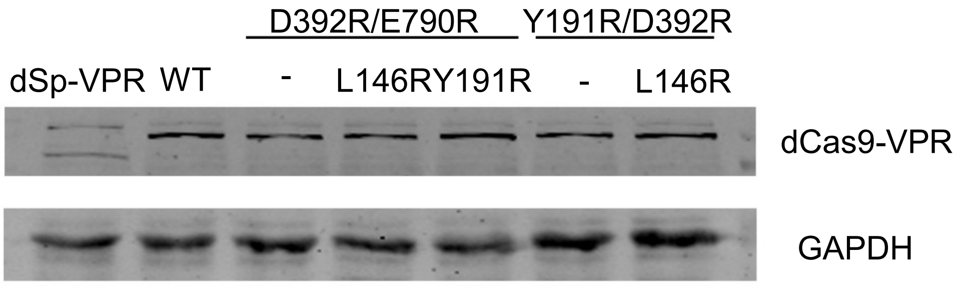

Figure S2. Western blot analysis of CjCas9 variant protein levels in HEK293T cells. The lanes are arranged from left to right as follows: SpCas9-VPR, WT, D392R/E790R, D392R/E790R/L146R, D392R/E790R/Y191R, Y191R/D392R, and Y191R/D392R/L146R CjCas9-VPR.

Figure S3

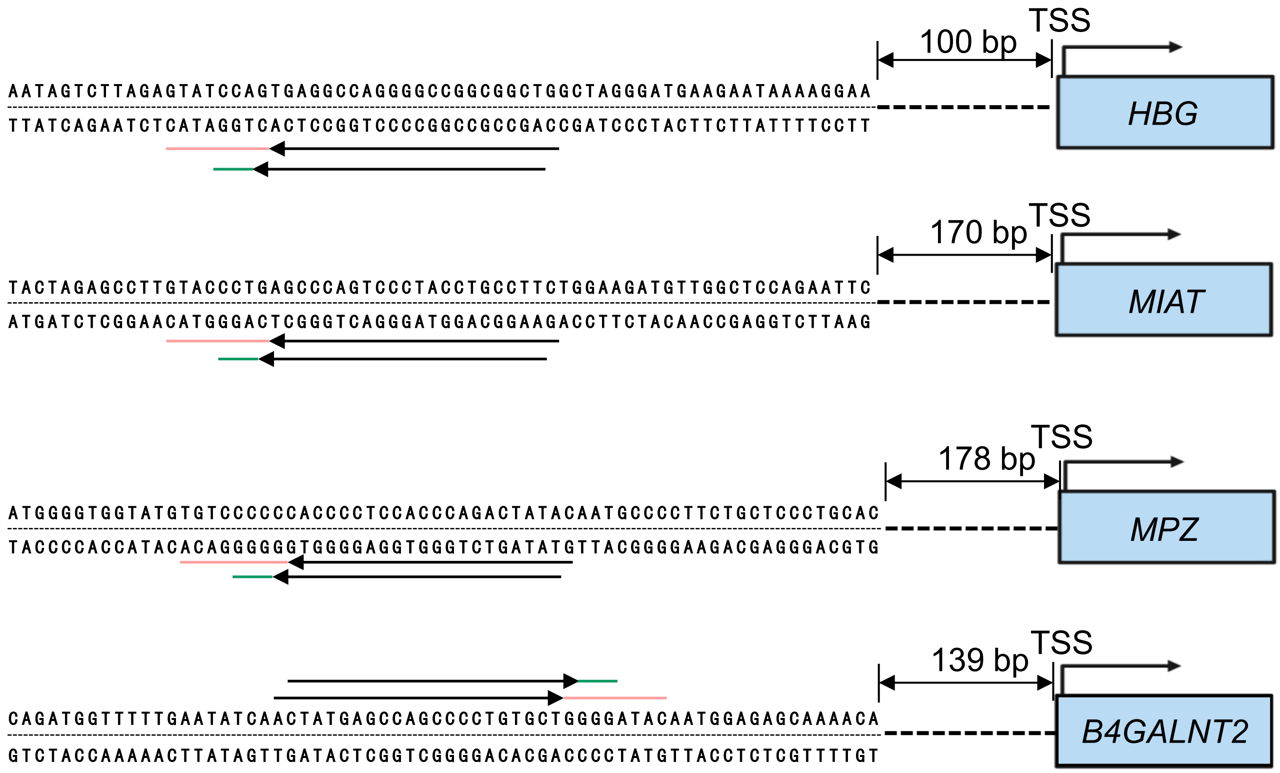

Figure S3. A schematic illustrating the sgRNAs of CjCas9 variants and SauriCas9 targeting regions upstream of the TSS for the *B4GALNT2, MIAT, HBG,* and *MPZ*. The orientation of the arrows signifies the direction of the 5′ to 3′ sequence of the spacer within the sgRNAs. The PAM motifs are distinguished by color-coded lines, with the CjCas9 variant depicted in pink and SauriCas9 in green referring to Figure 2C. TSS: transcription start site.

Figure S4

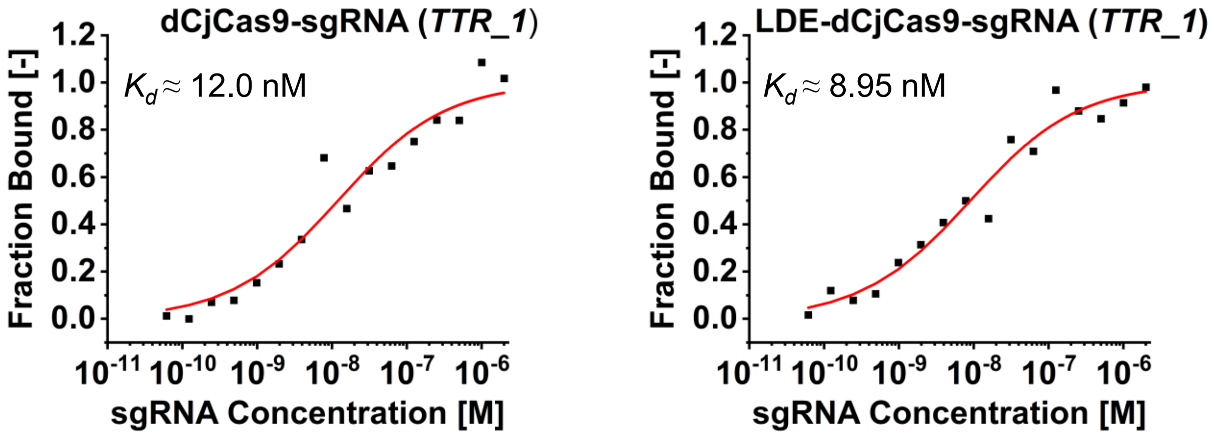

Figure S4. Microscale thermophoresis (MST) analysis of dCjCas9 or LDE-dCjCas9 protein binding to TTR sgRNA. The dissociation constants (Kd values), indicative of the strength of the protein-sgRNA interaction, were determined to be 12.0 nanomolar (nM) for dCjCas9 and 8.95 nanomolar (nM) for the LDE-dCjCas9 variant, respectively.

Figure S5

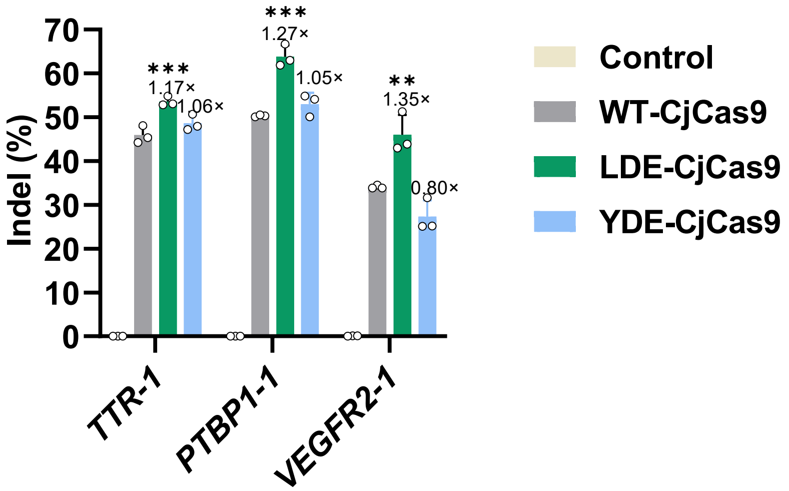

Figure S5. The indel frequencies induced by WT-CjCas9, LDE-CjCas9, and YDE-CjCas9 at the endogenous *TTR-1, PTBP1-1,* and *VEGFR2-1* target sites in HEK293T cells. Control: GFP. Fold changes comparing to wild type CjCas9 were shown on the top of each bar. Data represented three biological repeats and presented as mean ± S.E.M. Statistical significance was determined using two-tailed t test (*P < 0.05, **P < 0.01, ***P < 0.001)

Figure S6

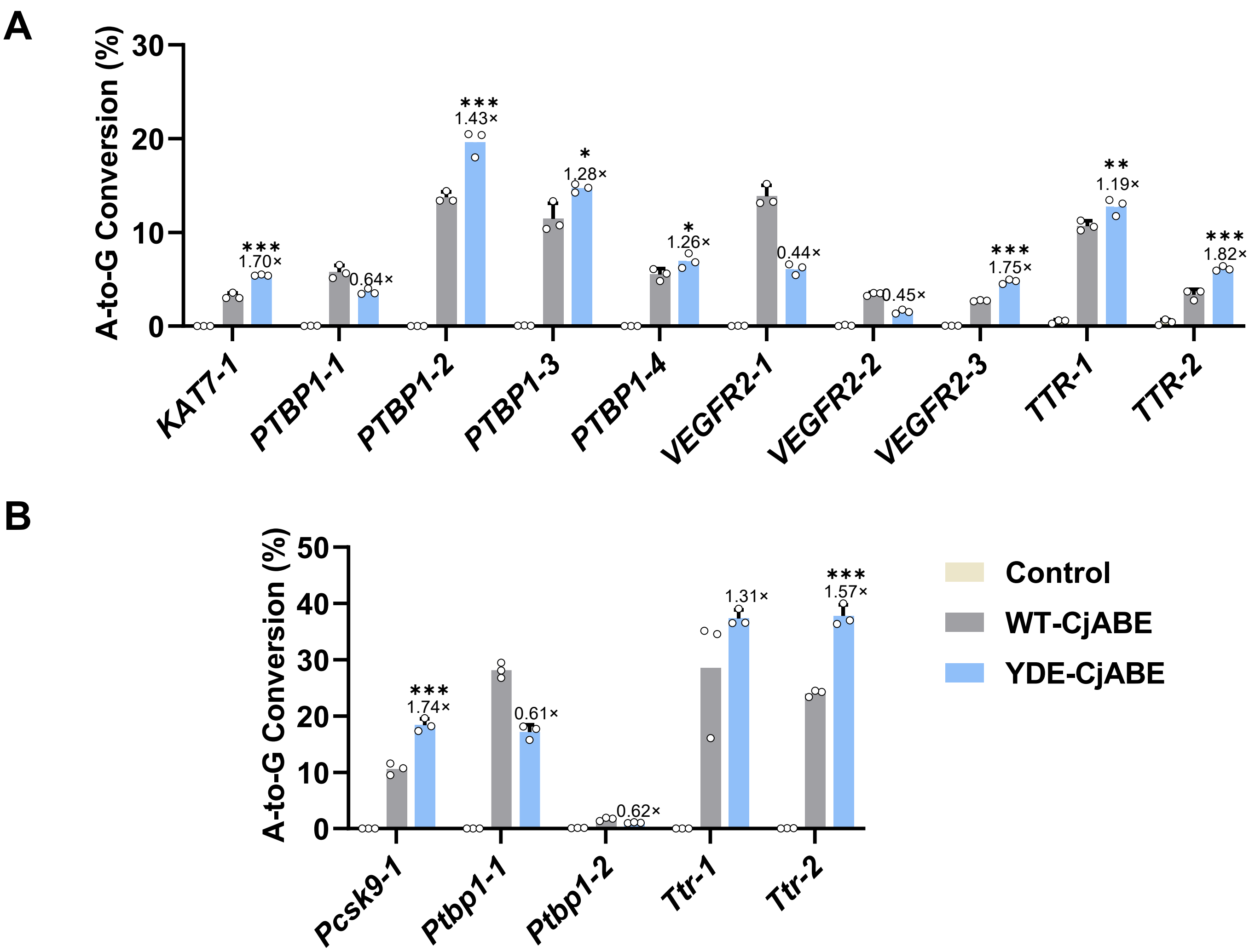

Figure S6. A-to-G editing efficiency of YDE-CjABEs. (A) A-to-G base editing induced by CjABE variants at the endogenous *KAT7, PTBP1, VEGFR2,* and *TTR* target sites in HEK293T cells. (B) A-to-G base editing induced by CjABE variants at the endogenous *Pcsk9, Ptbp1,* and *Ttr* target sites in mouse Hepa1-6 cells. Fold changes comparing to wild type CjCas9 were shown on the top of each bar. Data represented three biological repeats and presented as mean ± S.E.M. Statistical significance was determined using two-tailed t test (*P < 0.05, **P < 0.01, ***P < 0.001)

Figure S7

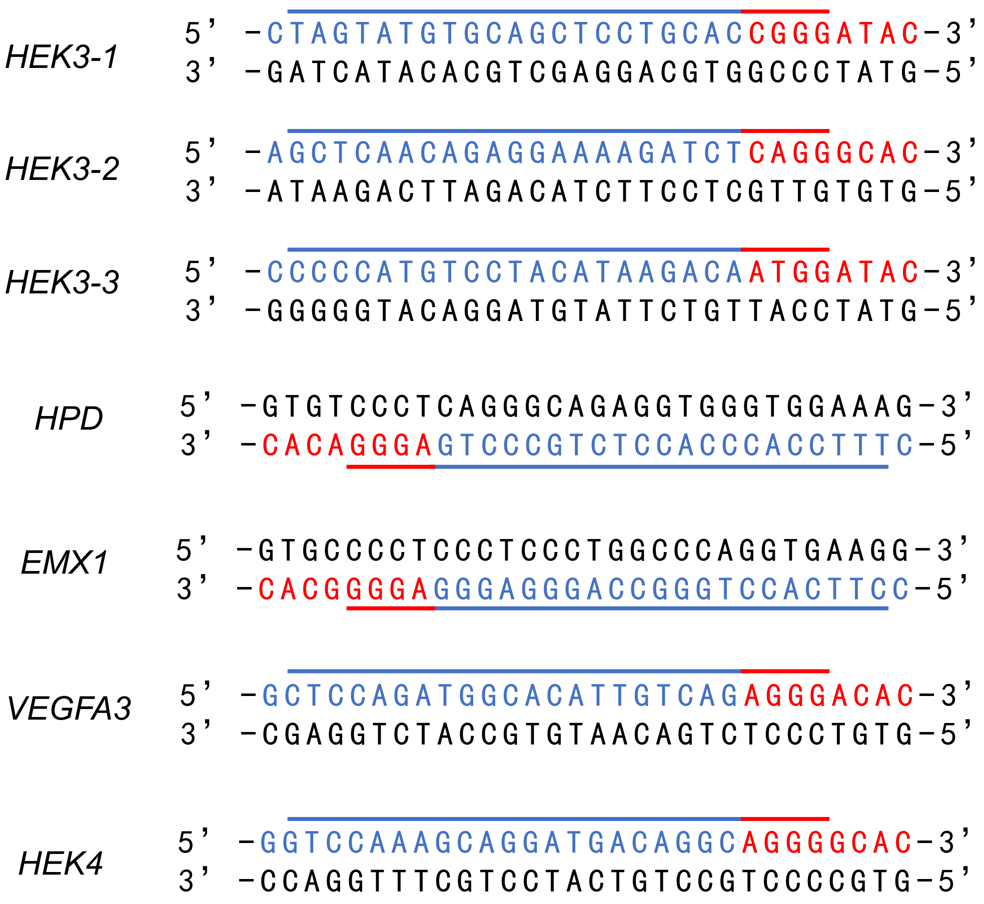

Figure S7. A schematic illustrating the sgRNAs of CjCas9 variant and SauriCas9 targeting for the *HEK3, HEK4, HPD,EMX1,* and *VEGFA3*. The sequence in red represents PAM (Protospacer Adjacent Motif), while the blue represents the spacer referring to Figure 3D. For the CjCas9s, the spacer is directly annotated on the sequence, and for SauriCas9, the spacer is denoted with a horizontal line.

Figure S8

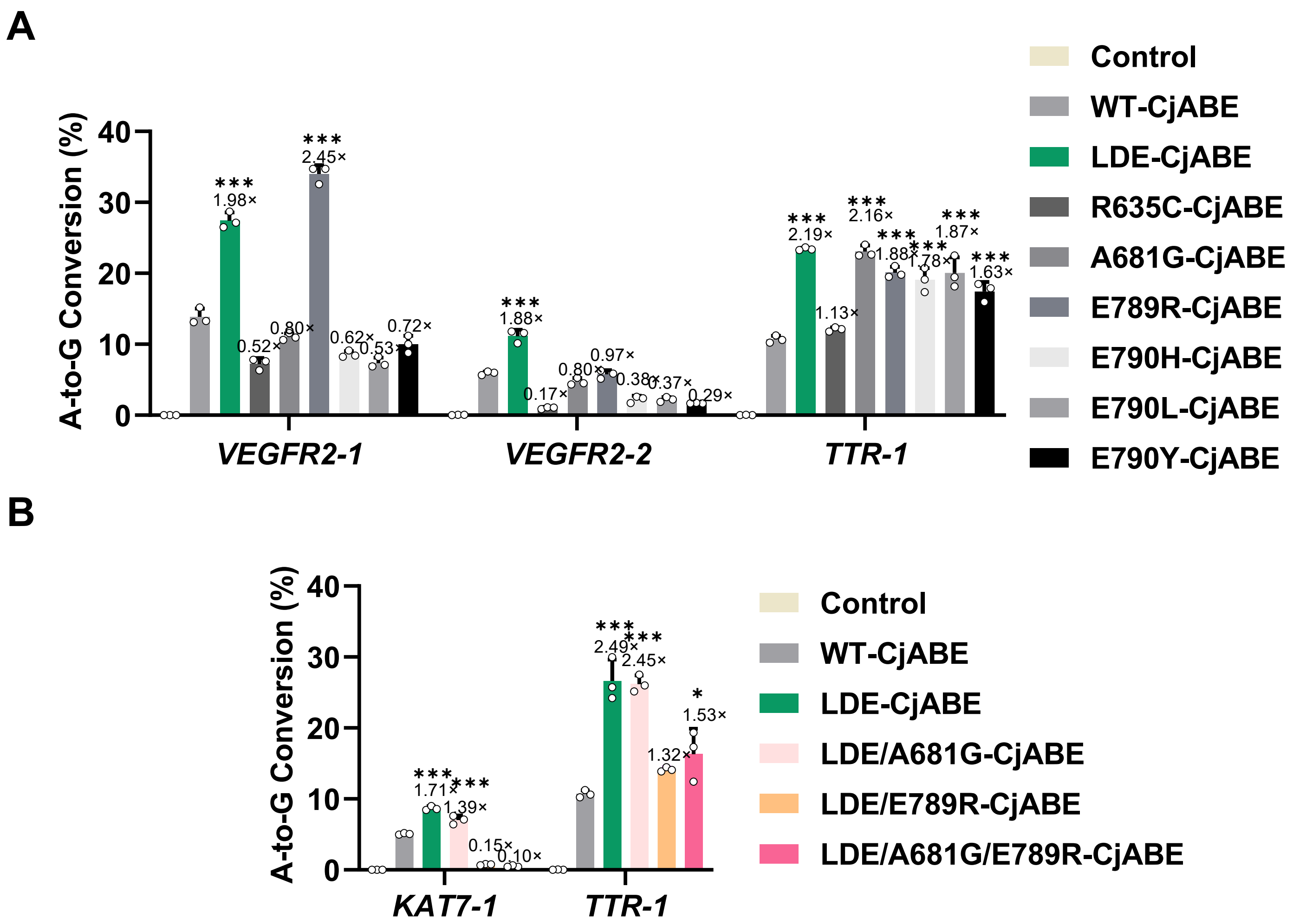

Figure S8. Installing the mutations of AceCas9 to the LDE-CjCas9. (A) A-to-G conversion frequencies of CjABEs at the endogenous *TTR* and *VEGFR2* target sites in HEK293T cells. (B) A-to-G conversion frequencies of CjABEs at the endogenous *KAT7* and *TTR* target sites in HEK293T cells. Control: GFP. Fold changes comparing to wild type CjCas9 were shown on the top of each bar. Data represented three biological repeats and presented as mean ± S.E.M. Statistical significance was determined using one-way ANOVA (*$P < 0.05$, **$P < 0.01$, ***$P < 0.001$)

Figure S9

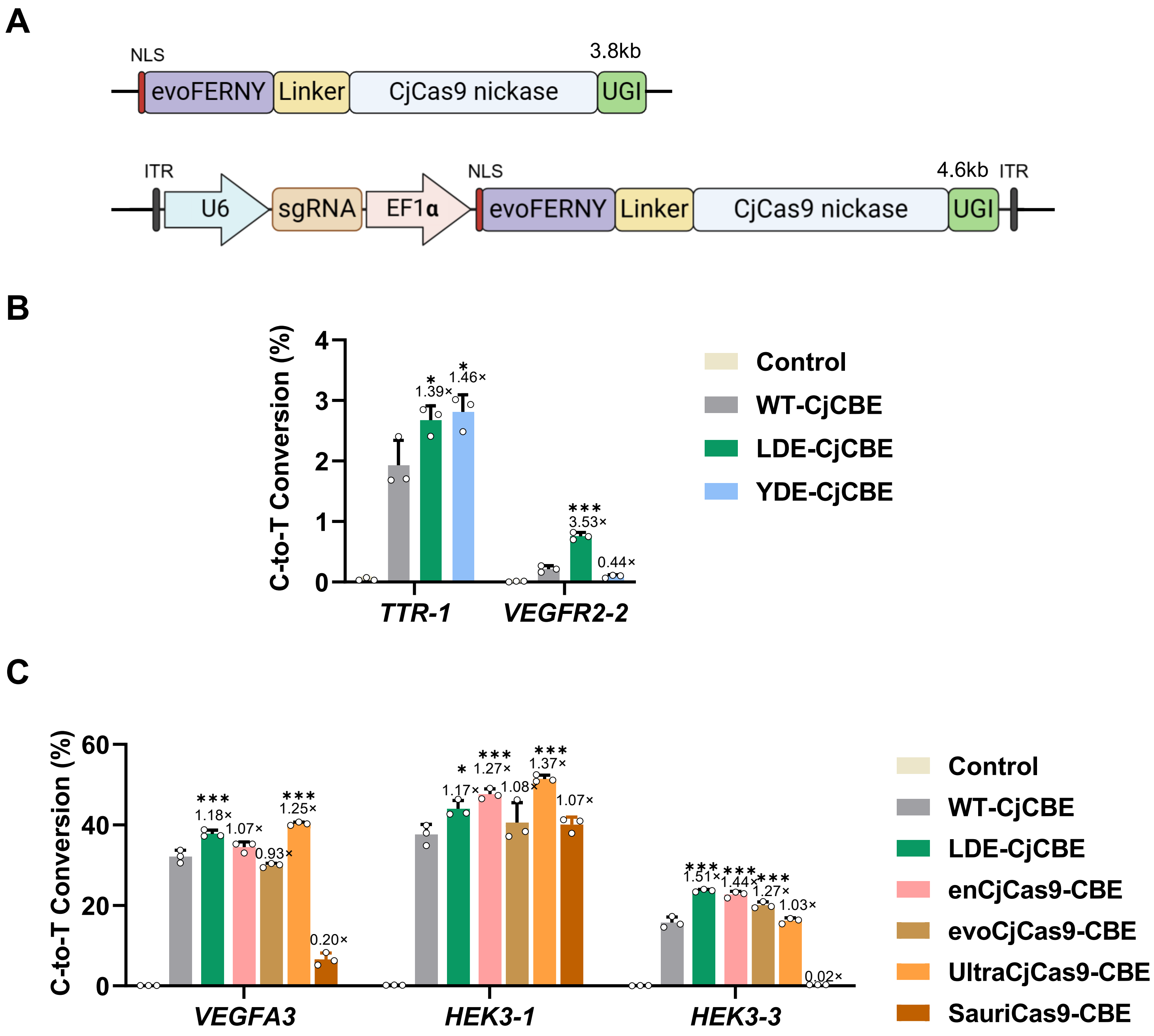

Figure S9. LDE-CjCBE with enhanced gene-editing C-to-T editing efficiency. (A) Schematic overviews of CjCBE constructs. (B) C-to-T conversion frequencies of CjCBEs at the endogenous *TTR* and *VEGFR2* target sites in HEK293T cells. (C) C-to-T conversion frequencies of CjCBE variants and SauriCas9-CBE at the endogenous *VEGFA3* and *HEK3* target sites in HEK293T cells. Control: GFP. Fold changes comparing to wild type CjCas9 were shown on the top of each bar. Data represented three biological repeats and presented as mean ± S.E.M. Statistical significance was determined using one-way ANOVA (*$P < 0.05$, **$P < 0.01$, ***$P < 0.001$)

Figure S10

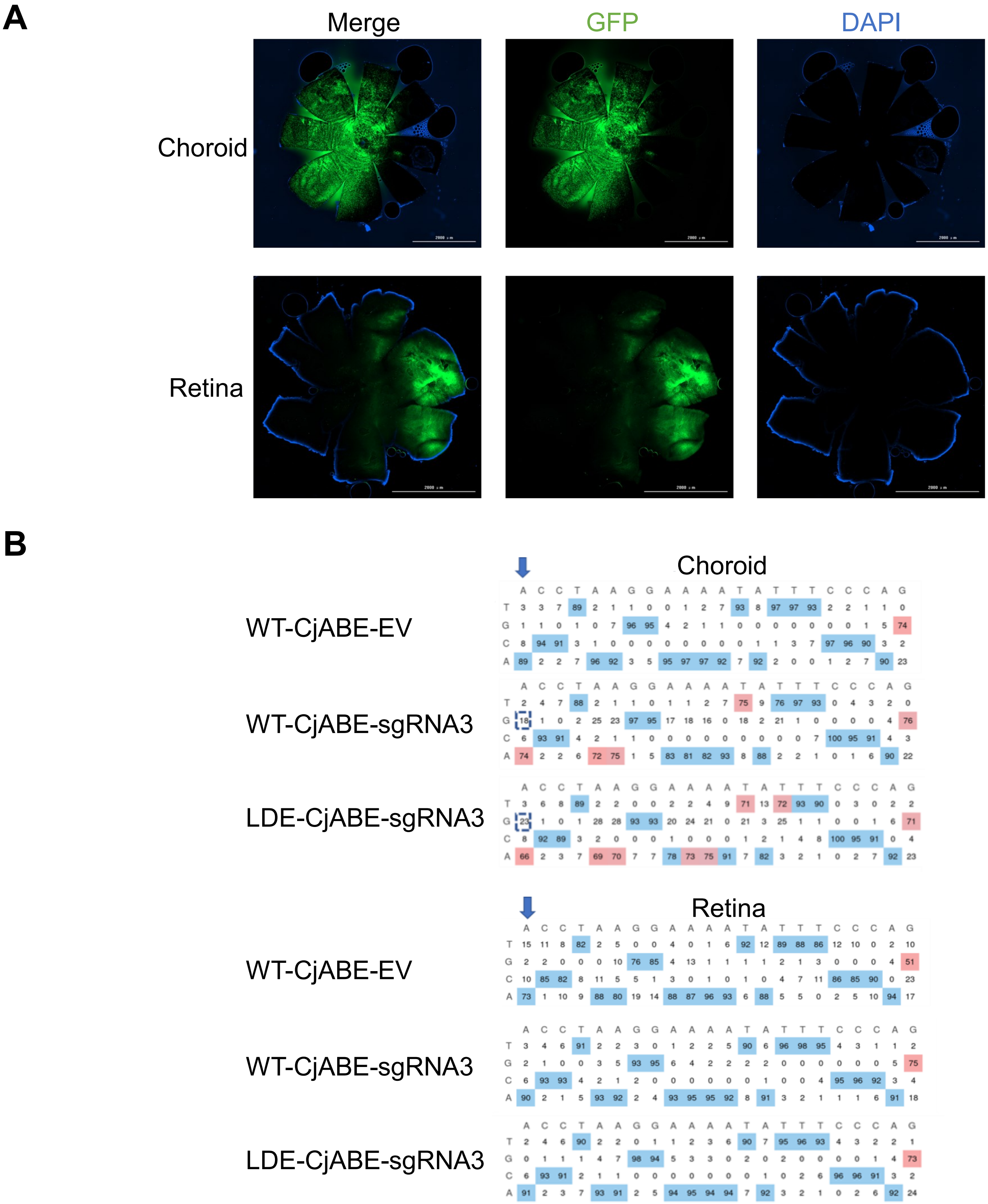

Figure S10. In vivo A-to-G editing efficiency in mouse retina and choroid two weeks post-injection. (A) Representative choroid and retinal flat-mount displaying GFP intensity. Scale bar: 2,000 μm. (B) Targeted A-to-G editing efficiencies of AAV-CjABE at the *Vegfr2-3* in mouse retina and choroid two weeks post-injection. Blue represents the type of base at the site, red indicates the possible base substitutions, and the dashed box represents the target base.

Table S1. CjCas9 amino acids within 3Å of the DNA backbone

Position	Amino Acid	Charge
131	Tyrosine	neutral
145	Isoleucine	neutral
146	Leucine	neutral
191	Tyrosine	neutral
277	asparagine	neutral
392	Aspartic acid	Negatively charged
790	Glutamic acid	Negatively charged
791	Threonine	neutral

Table S2. Comparison of the protein sequences and 3D structure of AceCas9 with CjCas9.

AceCas9	Mutation	AceCas9 3D Structure	CjCas9	Mutation	CjCas9 3D Structure
Arg671	R671C	-	Arg635	R635C	-
Val709	V709G	>4Å	Ala681	A681G	>4Å
Glu839	E839R	3.2-3.9Å	Glu789	E789R	3.2-3.9Å
Glu840	E840H	3.2-3.9Å	Glu790	E790H	3.2-3.9Å
Glu840	E840L	3.2-3.9Å	Glu790	E790L	3.2-3.9Å
Glu840	E840Y	3.2-3.9Å	Glu790	E790Y	3.2-3.9Å

Note: The 3D structural distance represents the distance from an amino acid to DNA.

Table S3. Potential off-target sites predicted by Cas-OFFinder for *Vegfr2_3*.

Off target site	chromosome	position	direction	mismatched base	DNA sequences
On-target	chr5	75944268	+	0	ACCTAAGGAAAATATTTCCCAGAGCAACAC
OT1	chr5	147578322	+	4	ACCTAAGGAgAAgATcTCCCAcAGCAACAC
OT2	chr1	24092659	+	4	ACagAAGtAAAATATTTCCCtGCTAAGCAC
OT3	chr1	130074038	-	4	ACCTAtGGcAAATATTgCCCAtTTTGATAC
OT4	chr12	91657775	+	4	ACCTAgGtAAAccATTTCCCAGAAGAGTAC
OT5	chr17	20342614	+	4	ACCTAAaGAAAATggTgCCCAGGAACATAC
OT6	chr16	50636960	+	4	ACCTAAaGAAAATAgTcCtCAGCAGCACAC
OT7	chr13	36014848	+	4	ACCTAtGGAAgATATTTCtCAtTCAAACAC
OT8	chr18	13287300	-	4	caCTAAGGAAgATcTTTCCCAGGCAGGCAC

Table S4. CjCas9-VPR target sequences.

Target name	Sequence (5' to 3')	PAM
TTN-1	GGAGAGGACCTATTTGGTAGTG	TCACATAC
TTN-2	TCAATCAGAAGAACAGGTGGTA	TGTGACAC
TTN-3	ACTGCCCACAAGTGCTATCTAC	TGTCACAC
HBG	CAGCCGCCGGCCCCTGGCCTCA	CTGGATAC
MIAT	GAAGGCAGGTAGGGACTGGGCT	CAGGGTAC
MPZ	TATAGTCTGGGTGGAGGGGTGG	GGGGACAC
B4GALNT2	ACTATGAGCCAGCCCCTGTGCT	GGGGATAC

Table S5. CjABE and CjCBE target sequences in HEK293T and Hepa 1-6.

Target name	Sequence (5' to 3')	PAM
TTR-1	AACACTCACCGTAGGGCCAGCC	TCGAACAC
TTR-2	CACCGTAGGGCCAGCCTCAGAC	ACAAATAC
Ttr-1	ATCACTCACCGCGGGGCCAGCT	TCAGACAC
Ttr-2	CACCGCGGGGCCAGCTTCAGAC	ACAAATAC
VEGFR2-1	GGAGATGCAACTTACTGCACAA	AGTGACAC
VEGFR2-2	GTTACTCACCTCCTGGCTTGGT	ACAGGCAC
VEGFR2-3	AGGTACCAGACCATGCTGGACT	GCTGGCAC
PTBP1-1	TCGCCGTCTGCAGGACCCACCT	GCCCGCAC
PTBP1-2	CAGCATTGTCCCAGATATAGCC	GTTGGTAC
PTBP1-3	TCCCCAACAGGCCTTCATCGAG	ATGAACAC
PTBP1-4	TTGCTCTGCTGTCTCTAAAGAG	AGTCACAC
Ptbp1-1	CAGCATCGTCCCAGACATAGCA	GTCGGTAC
Ptbp1-2	TTCCTCTGCTGTCTCTAAAGAG	AGTCACAC
KAT7-1	CTAGGAACACAGACAGACCTAT	GGGAACAC
Pcsk9-1	TAGGCCCTGAAGTTGCCCCATG	TGGAGTAC
HEK3-1	CTAGTATGTGCAGCTCCTGCAC	CGGGATAC
HEK3-2	AGCTCAACAGAGGAAAAGATCT	CAGGGCAC
HEK3-3	CCCCCATGTCCTACATAAGACA	ATGGATAC
HEK4	GGTCCAAAGCAGGATGACAGGC	AGGGGCAC
EMX1	CCTTCACCTGGGCCAGGGAGGG	AGGGGCAC
VEGFA3	GCTCCAGATGGCACATTGTCAG	AGGGACAC
HPD	CTTTCCACCCACCTCTGCCCTG	AGGGACAC

Table S6. AAV-CjABE target sequences in Hepa 1-6 and mouse retina.

Target name	Sequence (5' to 3')	PAM
Vegfr2-1	AACGAGGTAACTCACAGCGCAA	AGAGACAC
Vegfr2-2	TATTCTGAATCTGTAGAAGGAG	CAACACAC
Vegfr2-3	ACCTAAGGAAAATATTTCCCAG	AGCAACAC

Table S7. Primer sequences used in this study.

Sequencing primers	Sequence（5'-3'）
DS-H VEGFR2-g1-F	TGATTCCATGTCTCGGGTCC
DS-H VEGFR2-g1-R	ATTCATTGCATTTGACCCTTCCT
DS-H VEGFR2-g2-F	TATTGGTCACCATCTCAATGTGG
DS-H VEGFR2-g2-R	CCAGAGGAGTTGACTGCTTTCC
DS-H VEGFR2-g3-F	AAGTGCTTATTTCAGCATTCAGGA
DS-H VEGFR2-g3-R	ACCTGCTGAGCATTAGCTTGC
DS-H Kat7-g1-F	TGTGGTTTGCATGGATTGAAATG
DS-H Kat7-g1-R	GCTTGTGCTCTTCGGAAAAGAT
DS-H TTR-g1/g2-F	CTTGGCAGGATGGCTTCTCA
DS-H TTR-g1/g2-R	TTGGCAAAGCTGGAAGGAGT
DS-H PTBP1-g1-F	CGTGCAGTCTGTGGTGTCA
DS-H PTBP1-g1-R	TCCTGCCTCACTCCGGG
DS-H PTBP1-g2-F	ACCTACGGGCTCTCCTGG
DS-H PTBP1-g2-R	CAAGGTCAGAGGAGCGTCAC
DS-H PTBP1-g3-F	CCTGAGCCGCGTTTCTCC
DS-H PTBP1-g3-R	AGGGGTCACCGAGGTGTAG
DS-H PTBP1-g4-F	TTCCTCGTGTGGACGATTGG
DS-H PTBP1-g4-R	CTCAGGGCATCAAAATCATCTCA
DS-H HEK4-F	AGGGTCTGGAACCCAGGTAG
DS-H HEK4-R	GAGTCTCCGCTTTAACCCCC
DS-H HEK3-g1-F	GGGTGCCCTGAGATCTTTTCC
DS-H HEK3-g1-R	CTTCCTCCAGAGGGCGTC
DS-H HEK3-g2-F	GGGGCTAGTATGTGCAGCTC
DS-H HEK3-g2-R	CATGCAGGTGCTGAAAGCC
DS-H HEK3-g3-F	ACTTGTTACACGCAGGGCAC

DS-H HEK3-g3-R	CTTTGGGGTTTTCCAGCTGTT
DS-H VEGFA3-F	CCAAAGGACCCCAGTCACTC
DS-H VEGFA3-R	CCAAAGGACCCCAGTCACTC
DS-H EMX1-F	CTCAGTCTTCCCATCAGGCTC
DS-H EMX1-R	GCCCTTCTTCTTCTGCTCGG
DS-H HPD-F	CTTGCCTGGATATGAGGCCC
DS-H HPD-R	CAAGCACCCCTGAGGACTTC
DS-M TTR-g1、2-F	AGAAGCCGTCACACAGATCC
DS-M TTR-g1、2-R	AACTGCCATGTCTGGATCGC
DS-M PTBP1-g1-F	CCTGCCTTTCTAAGCCGGAC
DS-M PTBP1-g1-R	TGACTACAATGGGGTACAATGC
DS-M PTBP1-g2-F	TCTTCCTCGTGGACGATTGG
DS-M PTBP1-g2-R	ACCAATTAAAACGAAACCAAGGTA
DS-M VEGFR2-g1-F	GGCATCGTGTACATCACCGA
DS-M VEGFR2-g1-R	TGAAAGTGGGAAGCTCATCTCC
DS-M VEGFR2-g2-F	GTGGCAATGGGCACACTTAAT
DS-M VEGFR2-g2-R	GAGCAGATACTCACCTCCCG
DS-M VEGFR2-g3-F	GAGCGATGTGTGGTCTTTCG
DS-M VEGFR2-g3-R	ACAGGAGGTAACAGACCGAGA
DS-M PCSK9-g1-F	GAGGTGGACAGTCAGGTGG
DS-M PCSK9-g1-R	TGGGCGAAGACAAAGGAGTC
TTN qFP	TGTTGCCACTGGTGCTAAAG
TTN qRP	ACAGCAGTCTTCTCCGCTTC
GAPDH qFP	GGAGCGAGATCCCTCCAAAAT
GAPDH qRP	GGCTGTTGTCATACTTCTCATGG
MPZ qFP	CTCTCAGGTCACGCTGTATGT
MPZ qRP	GCAGTACCGAACCACGTAGAAA

B4GALNT2 qFP	CCTGCACACGGTTCCCATC
B4GALNT2 qRP	AGCAAGGGTGTTCAGTGTCC
HBG qFP	TGCACTGTGACAAGCTGCAT
HBG qRP	TGGATTGCCAAAACGGTCAC
MIAT qFP	GGTTGGCTCTTTTGTCTTCCAGG
MIAT qRP	GGCTCAGAGAAGTTGCTTGGTC
β-actin-PCR-F	GTCCCTCACCCTCCCAAAAG
β-actin-PCR-R	GCTGCCTCAACACCTCAACCC
AAV-PCR-F	TCCTTCCTGGTGGAAGAGGATAA
AAV-PCR-R	CTCGAACAGCTGGTTGTAGGTC
