# Supplementary material for: Rationally designed Campylobacter jejuni Cas9 enables efficient gene activation and base editing
Source: Mol Ther Nucleic Acids. 2024 Oct 18;35(4):102366. doi: 10.1016/j.omtn.2024.102366 (PMC11570936; doi:10.1016/j.omtn.2024.102366)
Supplement: Document S2. Article plus supplemental information [file mmc2.pdf]

Molecular Therapy
Nucleic Acids
Original Article

Rationally designed *Campylobacter jejuni* Cas9 enables efficient gene activation and base editing

Yuxi Chen,[1,5] Rui Kang,[1,5] Yuanling Jiang,[1,2,5] Qi Zheng,[1,5] Yue Yang,[1] Jiaqi Liu,[1] Guanglan Wu,[1] Weijun Zhao,[1] Zhan Li,[1] Chengxiang Peng,[1] Pengfei Zhang,[1] Fei Peng,[1] Qianyi Liu,[1] Sihui Hu,[1] Xiao Luo,[1] Guifang Wu,[1] Kaixin Cui,[1] Junjiu Huang,[1] Yongming Wang,[3,4] Zhou Songyang,[1] and Puping Liang[1]

[1]MOE Key Laboratory of Gene Function and Regulation, State Key Laboratory of Biocontrol, School of Life Sciences, Sun Yat-sen University, Guangzhou 510275, China; [2]Department of Cellular and Molecular Diagnostics Center, Sun Yat-sen Memorial Hospital, Sun Yat-sen University, Guangzhou 510000, China; [3]State Key Laboratory of Genetic Engineering, School of Life Sciences, Zhongshan Hospital, Fudan University, Shanghai 200438, China; [4]Shanghai Engineering Research Center of Industrial Microorganisms, Shanghai 200438, China

Compact and adaptable CRISPR-Cas systems enable genome engineering applications in various contexts via high-efficiency delivery. The adeno-associated virus (AAV) is a widely used delivery system. One of the most compact type II-C Cas9 orthologs—CjCas9, derived from *Campylobacter jejuni,* is particularly appealing for AAV delivery. However, the editing efficiency of CjCas9 limits its applications. In this study, we used structure-guided protein engineering to improve the editing efficiency of CjCas9. Subsequently, we developed a miniature transcriptional activator (LDE-CjCas9-VPR) and base editors engineered from CjCas9 (LDE-CjABE and LDE-CjCBE). LDE-CjABE effectively induced genome editing in human and mouse cells. Through AAV delivery, LDE-CjABE enhanced the on-target editing efficiency, and off-target editing was not detected in the mouse retina. Therefore, the compact size and high editing efficiency of LDE-CjCas9 broadens the target scope of transcription activation and base editing toolsets for therapeutic applications.

INTRODUCTION

The clustered regularly interspaced short palindromic repeats (CRISPR) system, which functions as an adaptive immune system in bacteria and archaea, has been developed into various genome-editing tools.[1] Because of its high editing effectiveness and the recognition of a relaxed 5′-NGG-3′ protospacer adjacent motif (PAM), *Streptococcus pyogenes* Cas9 (SpCas9) has become dominant in genome editing.[2] Base editors (BEs), comprising deaminases and Cas9 nickase, enable precise C-to-T (CBE) or A-to-G (ABE) conversions in genomic DNA, making them potent tools for therapeutic genome editing applications.[3–5]

However, the large size of these tools often hinders cellular delivery, thereby limiting their clinical application. For instance, adeno-associated virus (AAV), a widely used vector for *in vivo* delivery, has a payload packaging capacity of 4.7 kb, and many Cas fusion proteins exceed this limit. Solutions to this issue include, on the one hand, dividing the BEs into two smaller portions by intein-mediated protein *trans*-splicing and delivering them by a pair of AAV vectors.[6,7] On the other hand, several BEs based on small Cas9 orthologs (e.g., SauriCas9 and Cje3Cas9) had been developed for *in vivo* genome-editing therapy.[8,9] CjCas9 is 984 amino acids long and is among the most compact Cas9 proteins.[10–13] Moreover, BEs based on CjCas9 could be packaged into a single AAV. However, the editing efficiency of CjCas9 is relatively low in human cells, significantly limiting its applications.[14] Therefore, it is critical to enhance the editing capability of CjCas9.

Comparative analysis shows that, unlike the type II-A SpCas9, the smaller type II-C Cas9 proteins exhibit limited dsDNA binding and unwinding activity.[15] Previous studies have shown that the disruption of non-specific interactions between Cas9 and the phosphate backbone of target DNA weakens target DNA binding by the Cas9-single guide RNA (sgRNA) complex, leading to the development of improved Cas9 variants (eSpCas9, SpCas9-HF, and SaCas9-HF) with higher specificity.[16–18] We hypothesized that enhancing the interaction between CjCas9 and the phosphate backbone of the target DNA would improve editing efficiency. The crystal structure of CjCas9 in complex with guide RNA and target DNA provides a foundation for rational engineering to improve the editing capacity.[11,12] The structure indicates a positively charged groove between the HNH, RuvC, and PAM-interacting domains in CjCas9, which is likely to be involved in stabilizing the target DNA. We hypothesized that replacing negatively charged (or neutral) residues in this groove with positively charged residues would enhance the interaction between CjCas9 and target DNA, thereby improving its performance.

In this study, we created a series of nuclease-dead CjCas9 (dCjCas9, D8A, and H559A) variants that could efficiently activate endogenous

Received 24 April 2024; accepted 16 October 2024;
https://doi.org/10.1016/j.omtn.2024.102366.

[5]These authors contributed equally

Correspondence: Puping Liang, MOE Key Laboratory of Gene Function and Regulation, State Key Laboratory of Biocontrol, School of Life Sciences, Sun Yat-sen University, 132 East Waihuan Road, Guangzhou 510275, China.
E-mail: liangpp5@mail.sysu.edu.cn

© 2024 The Author(s).
Published by Elsevier Inc. on behalf of The American Society of Gene and Cell Therapy.
This is an open access article under the CC BY license (http://creativecommons.org/licenses/by/4.0/).

gene expression when fused with a transcriptional activator. In addition, the adenine base editor adapted from these CjCas9 variants (LDE-CjABE) allowed robust A-to-G conversion at endogenous sites. Furthermore, single AAV-mediated delivery of LDE-CjABE efficiently induced gene editing in the ocular retina of mice, with potential therapeutic implications. Importantly, CjCas9 variants maintained the specificity of CjCas9. Here, we applied protein engineering to improve the editing efficiency of CjCas9, and generated a compact, efficient, and specific system for mammalian genome engineering.

RESULTS

Screening CjCas9 variants with enhanced DNA binding activity

We propose that CjCas9-mediated DNA targeting can be improved by increasing the affinity of CjCas9 to target DNA.[19] We hypothesized that substituting the amino acids close to the DNA backbone phosphate with positively charged arginine would enhance the interaction between CjCas9 and target DNA. Lysine, histidine, and arginine are all positively charged amino acids, and arginine has a longer side chain that is more flexible to contact with negatively charged DNA backbone phosphate. Therefore, we substituted these amino acids with arginine. To avoid altering the PAM preference of engineered CjCas9, we examined its protein structure outside the PAM-interacting domain (PID) and identified eight amino acids within 3Å of the DNA backbone (Figures 1A and S1; Table S1).

To test our hypothesis, we generated dCjCas9-VPR (nuclease-dead CjCas9 [D8A and H559] fused with VP64-P65-RTA) to assess the interaction between CjCas9 variants and target DNA. We performed a screening assay using a previously reported GFP reporter system driven by dCjCas9-VPR,[20,21] in which the GFP cassette was driven by a minimal CMV promoter. Using this reporter and a CMV promoter targeting guide RNA, the binding ability of engineered dCjCas9 to the minimal CMV promoter could be indicated by the activation of GFP (Figure 1B). Plasmids encoding dCjCas9-VPR and its eight variants were transiently transfected into the reporter cells. GFP percentage of GFP-positive cells was quantified by flow cytometry 3 days post-transfection, with dSpCas9-VPR as a positive control. The mean percentage of GFP-positive cells in dCjCas9-VPR was 0.40%, indicating the weak binding ability of wild-type dCjCas9-VPR to the target DNA. Although some engineered dCjCas9-VPR variants displayed activity similar to or lower than that of the wild-type (WT) protein, three single-point mutations, Y191R, D392R, and E790R, increased the percentage of GFP-positive cells (Figure 1C).

Next, we combined the beneficial mutations (Y191R, D392R, and E790R) to boost the DNA binding ability of engineered dCjCas9-VPR. We found that Y191R/D392R and D392R/E790R significantly increased the percentage of GFP-positive cells (Figure 1D). We generated triple-mutant variants based on these two dual-mutant variants (Y191R/D392R and D392R/E790R) (Figure 1E). The percentage of GFP-positive cells was significantly increased in the L146R/D392R/E790R and Y191R/D392R/E790R variants compared with that in dCjCas9-VPR (Figure 1E). The top-performing variant, L146R/D392R/E790R (referred to as LDE [LDE-CjCas9] in the latter text), achieved a 15.99-fold enhancement in the percentage of GFP-positive cells compared with dCjCas9-VPR (Figure 1E) without altering the protein level (Figure S2). These findings suggest that substituting L146/D392/E790 in CjCas9 (LDE-dCjCas9) with positively charged arginines may significantly boost the affinity of CjCas9 to the target DNA.

LDE-dCjCas9-VPR activated endogenous gene expression in mammalian cells

To explore the feasibility of activating endogenous gene expression by CRISPR activation (CRISPRa) tools based on CjCas9, we evaluated the activating levels of endogenous genes by dCjCas9-VPR, LDE-dCjCas9-VPR, and dSpCas9-VPR in HEK293T cells. sgRNAs targeting the transcription start sites (TSS) of *TTN* were designed at −300 base pairs (bp) to −3 bp upstream of the TSS (Figure 2A). To compare the activation efficiencies, we designed dSpCas9 sgRNAs overlapping with dCjCas9 sgRNAs as controls. At all three sites tested, LDE-dCjCas9-VPR outperformed dCjCas9-VPR (Figure 2B). The activation efficiency of LDE-dCjCas9-VPR was comparable to that of dSpCas9-VPR at sgRNA1 and sgRNA3 target sites (Figure 2B).

To provide a comprehensive understanding of current existing engineered CjCas9 variants, we conducted a comparative transcription activating analysis of LDE-CjCas9 alongside with other engineered CjCas9 variants and small Cas9 orthologs including enCjCas9,[22] evoCjCas9,[23] UltraCjCas9,[24] and SauriCas9.[8] All Cas9 variants were cloned into a uniform backbone vector to ensure fair comparison and minimize bias. Additionally, we designed guide RNAs (gRNAs) with identical spacer sequences to maintain consistency across experiments (Figure S3). Performance of different engineered Cas9s varied among different targets (Figure 2C). This variability in transcription activating level might be, in some degree, due to the sequence preferences of different engineered Cas9 variants.[25] Therefore, selection of the most suitable CjCas9 variant is important when targeting a specific target. These results proved that CRISPRa based on LDE-dCjCas9 could activate endogenous gene expression, suggesting that LDE-dCjCas9-VPR had higher activity than dCjCas9-VPR in activating endogenous genes.

To dissect the binding affinity of the CjCas9-sgRNA ribonucleoprotein (RNP) complex with the target DNA, we utilized microscale thermophoresis (MST) to determine the dissociation constant (K_d) of the dCjCas9 variants. For binding affinity measurements, we purified dCjCas9 tagged with GFP from bacteria. Subsequently, we investigated the binding of the dCjCas9-sgRNA RNP complex with two different DNA substrates (*VEGFR2* and *TTR*), and the K_d values were 48.2 nM and 28.4 nM, respectively (Figure 2D). The K_d values of LDE-dCjCas9 to the two targets were 27.7 nM and 18.2 nM, respectively, suggesting that LDE-dCjCas9 had a higher affinity to target DNA. Moreover, compared with dCjCas9, LDE-dCjCas9 exhibits higher affinity to the sgRNA, and K_d values were 12.0 nM and 8.95 nM, respectively (Figure S4). Moreover, indel frequency induced by LDE-dCjCas9 also increased slightly in HEK293T cells (Figure S5). Collectively, these data suggest that the affinity of LDE-dCjCas9 with DNA was higher than that of WT CjCas9.

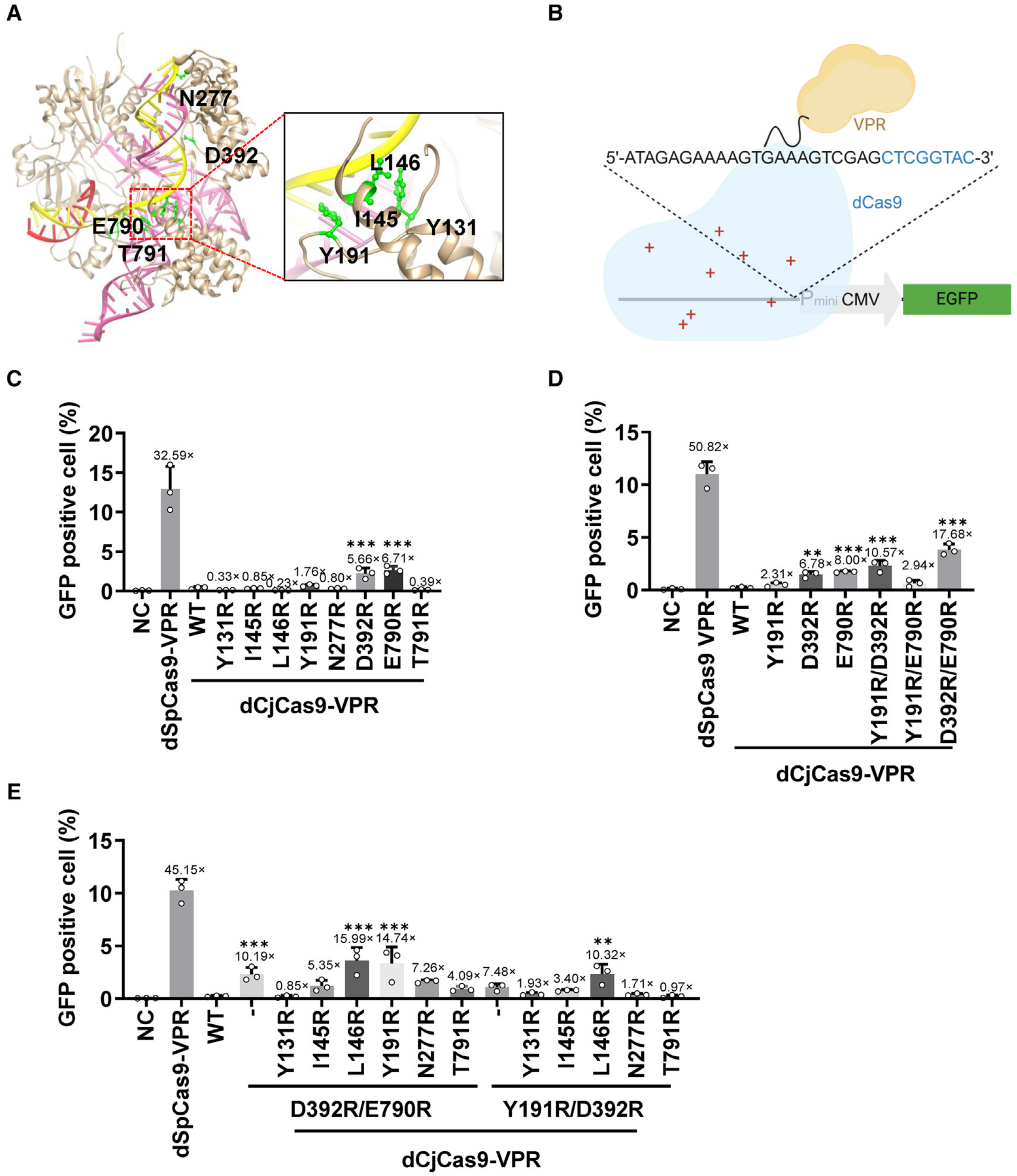

Figure 1. Enhancement of DNA binding by engineering CjCas9

(A) The protein structure of CjCas9 with mutations introduced in this study (based on PDB ID 5X2H). (B) A schematic illustrating the integrated sequence in the GFP reporter cell, highlighting the CjCas9 target sequences. The PAM sequence is blue, while the target sequence is black. PminiCMV, mini CMV promoter. (C–E) The percentages of GFP-positive cells induced by CjCas9 variants that bear one (C), two (D), and three (E) mutations. Control: GFP. WT: wild-type CjCas9. Fold changes compared with WT CjCas9 are shown on the top of each bar. Data represent three biological repeats and are presented as mean ± SEM. Statistical significance was determined using one-way ANOVA (*$p < 0.05$, **$p < 0.01$, ***$p < 0.001$).

The LDE variant improved the efficiency of the CjCas9 BEs

Next, we investigated whether the LDE mutant was compatible with other CjCas9-derived tools such as BEs. The two variants (L146R/D392R/E790R, referred to as LDE; Y191R/D392R/E790R, referred to as YDE) and CjCas9 nickase (nCjCas9, D8A) were fused with TadA-8e to construct three CjABEs suitable for single AAV delivery (cargo size <4.6 kb) (Figure 3A). We targeted 10 sites across four genes in HEK293T cells and performed deep sequencing to assess the editing efficiency of LDE-CjABE and YDE-CjABE (Figures 3B and S6). The LDE variants showed 2- to 3-fold increase in A-to-G

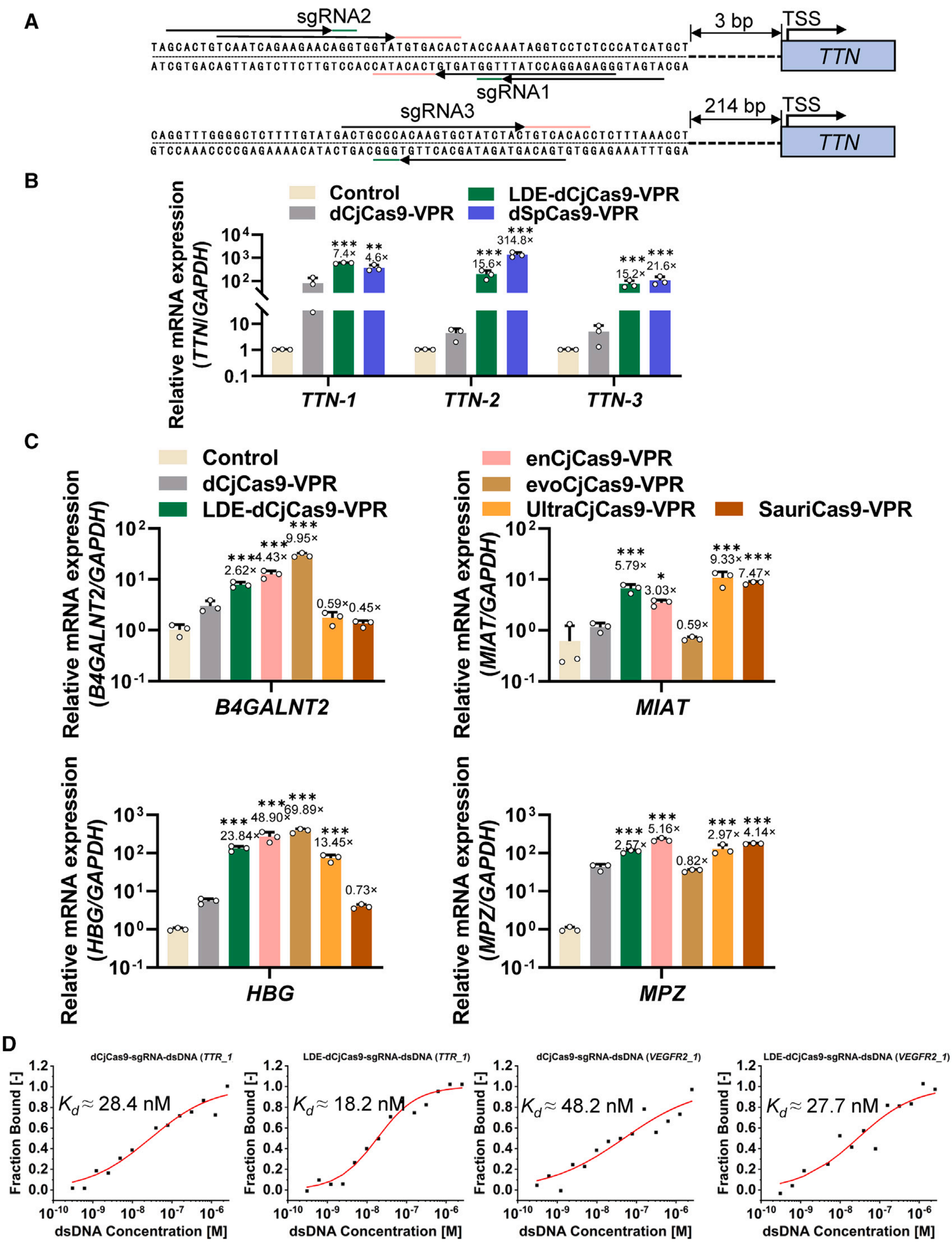

Figure 2. Transcriptional activation of endogenous genes by LDE-dCjCas9 in human cells

(A) A schematic illustrates the sgRNAs of dSpCas9 and dCjCas9 targeting different regions upstream of the TSS of *TTN*. The direction of the arrows indicates the 5′–3′ sequence of the spacer. The colored lines (green and pink) represent the PAM motif. TSS: transcription start site. (B) Quantitative PCR result of *TTN* transcription levels in HEK293T cells transfected with dSpCas9-VPR, dCjCas9-VPR, or LDE-dCjCas9-VPR. Control: GFP. (C) Quantitative PCR results of *B4GALNT2, MIAT, HBG,* and *MPZ* transcription levels in HEK293T cells transfected with various transcription activators including dCjCas9-VPR, LDE-dCjCas9-VPR, enCjCas9-VPR, evoCjCas9-VPR, UltraCjCas9-VPR, and SauriCas9-VPR. Control: GFP. (D) MST analysis of WT-dCjCas9 or LDE-dCjCas9 protein with *TTR* or *VEGFR* targets. Fold changes compared with wild-type CjCas9 are shown on the top of each bar. Data represent three biological repeats and are presented as mean ± SEM. Statistical significance was determined using one-way ANOVA (*$p < 0.05$, **$p < 0.01$, ***$p < 0.001$).

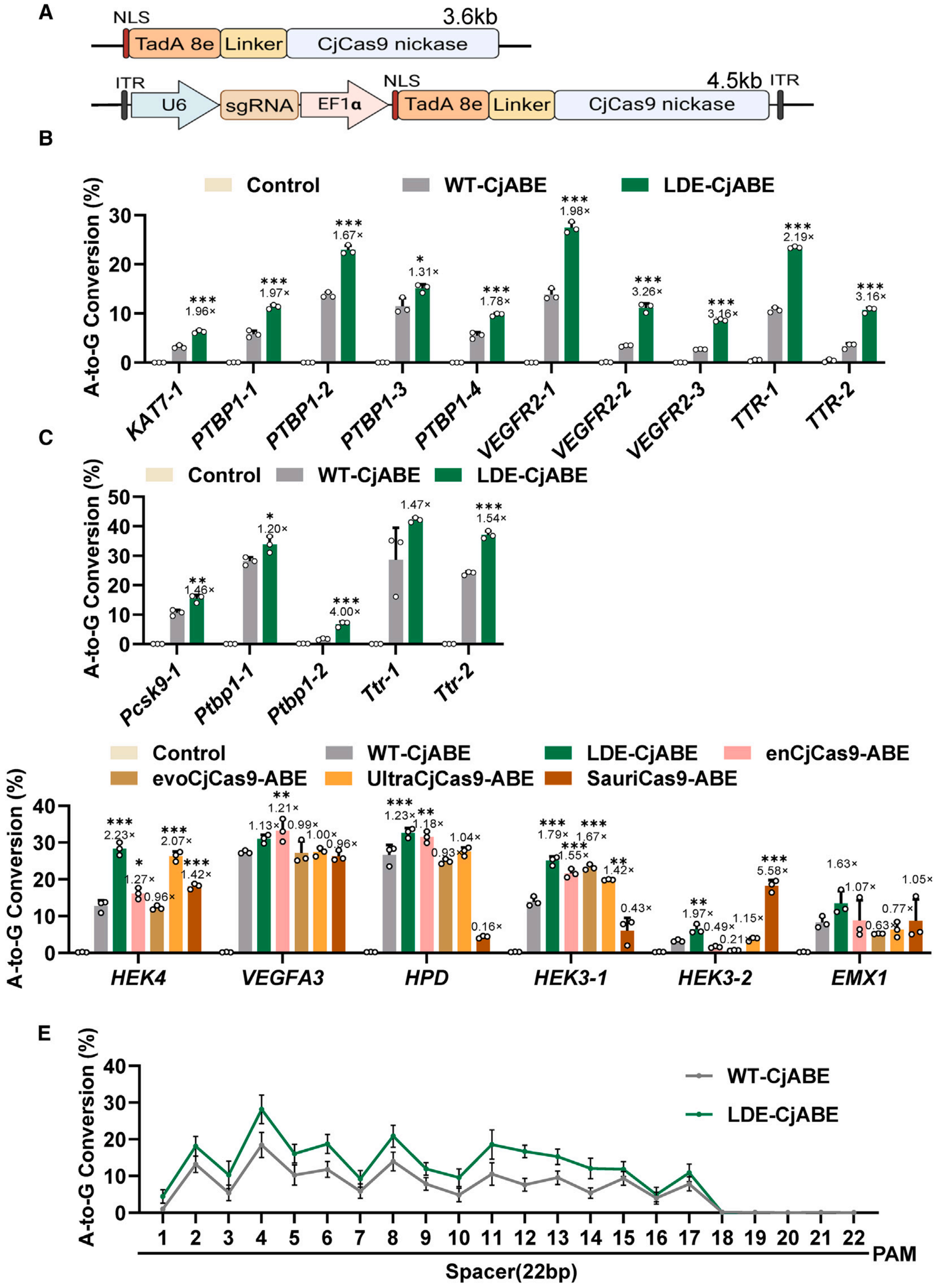

Figure 3. LDE-CjABE enhanced A-to-G editing efficiency

(A) Schematic overviews of CjABE and all-in-one CjABE AAV constructs. (B) A-to-G conversion frequencies of CjABEs at the endogenous *KAT7, PTBP1, VEGFR2,* and *TTR* target sites in HEK293T cells. (C) A-to-G conversion frequencies of CjABEs at the endogenous *Pcsk9, Ptbp1,* and *Ttr* target sites in mouse Hepa1-6 cells. (D) A-to-G conversion frequencies of WT-CjABE, LDE- CjABE, enCjCas9-ABE, evoCjCas9-ABE, UltraCjCas9-ABE, and SauriCas9-ABE at the endogenous *HEK3, HEK4, EMX1,*

(legend continued on next page)

editing efficiency compared with WT at all sites tested. However, the YDE-CjABE did not show consistent enhancement over different targets (increased at seven sites and decreased at three sites) (Figures 3B and S6A). Next, we selected five targets in Hepa1-6 cells to test the performance of LDE and YDE-CjABE in mouse cell lines. Similarly, LDE-CjABE exhibited higher A-to-G editing efficiency than WT-CjABE at all five target sites (Figure 3C). In contrast, the editing efficiency improvements of YDE-CjABE were observed in only two out of five target sites (Figure S6B).

We subsequently evaluated the A-to-G base editing efficiency of LDE-CjABE compared with that of other engineered CjCas9s and SauriCas9 targeting the same genomic loci (Figures 3D and S7). Our analysis encompassing six genomic loci revealed no significant superiority in adenosine base editing efficiency among the variants (Figure 3D), again reflecting the sequence preferences of different engineered Cas9s in targeted gene editing.[25] To characterize the base editing window of LDE-CjABE, we analyzed the A-to-G editing frequencies among all the target As in the 22-nt spacers. We found that CjABE had a wide editing window (A1-17), and LDE-CjABE exhibited higher activity within the editing window (Figure 3E).

Through bacterial toxicity survival experiments, the nuclease activity of AceCas9 (*Acidothermus cellulolyticus* Cas9), another type II-C Cas9 protein, was improved.[26] AceCas9 and CjCas9 are both type II-C Cas9 and share a high homology.[26] Their bridge helix and RuvC, REC, HNH, WED, and PI domain arrangements exhibited a high degree of similarity.[27] Inspired by previous work showing that a combination of different mutations from Cas9 variants may increase activity,[28] we mapped the beneficial mutations of AceCas9 to CjCas9 to try further improving the editing efficiency of CjCas9. By aligning the sequences of AceCas9 and CjCas9, we then assessed whether the editing capabilities of CjABE could be improved by introducing R635C, A681G, E789R, E790H, E790L, and E790Y mutations into CjCas9 (Table S2). The editing efficiency of A-to-G for the variants A681G and E789R was improved compared with that of WT-CjABE (Figure S8A). We wanted to explore whether combining A681G and E789R with the LDE variant would further enhance the efficiency. However, adding these two single mutations or the dual-mutation on the LDE did not improve the editing efficiency (Figure S8B).

In addition, we fused evoFERNY,[29] a smaller cytidine deaminase, with nCjCas9s and uracil glycosylase inhibitor (UGI) to generate CjCBE that could be packaged into a single AAV vector (Figure S9A). The C-to-T editing efficiency of CjCas9-CBE was relatively low (TTR-1 site: ∼2%, VEGFR2-2 site: 0.3%); however, LDE-CjCBE showed increased C-to-T editing efficiency at these sites (TTR-1 site: ∼2.5%, VEGFR2-2 site: 0.9%) (Figure S9B). LDE-CjCBE and other engineered CjCas9 variants showed nearly the same improvement in C-to-T editing efficiency compared with WT CjCas9 (Figure S9C). Collectively, these data demonstrated that the LDE variant could increase the editing efficiency of BEs based on CjCas9 nickase *in cellulo*.

Single AAV delivery of LDE-CjABE achieved efficient and specific editing in mouse retina

Age-related macular degeneration (AMD) is a chronic degenerative eye disease that primarily affects the macular area of the retina. It has a high incidence and ranks third among the leading causes of blindness worldwide. Wet AMD (wAMD) is characterized by the development of choroidal neovascularization (CNV) between the retina and choroid, leading to retinal exudation, bleeding, and central vision loss. As the VEGFA-VEGFAR2 pathway is highly related to CNV, to assess the feasibility of *in vivo* gene editing therapy using CjABE delivered by AAV, *Vegfr2* was chosen as the target to inhibit CNV (Figure 3A). Three sgRNAs targeting the *Vegfr2* mRNA splicing sites were designed for the mouse *Vegfr2* gene (Figure 4A). In mouse Hepa1-6 cells, LDE-CjABE exhibited a higher editing efficiency than WT-CjABE, especially at the sgRNA3 site (Figure 4B). Overall, the LDE-CjABE exhibited higher editing efficiency and was more suitable for disrupting the splicing of *Vegfr2* mRNA.

AAV8, known for its retinal tropism, is ideal for delivering CjABEs to mice via subretinal injection. AAV8-GFP and AAV8 WT-CjABE-sgRNA empty vectors (referred to as WT-CjABE-EV) were used as controls, while AAV8 WT-CjABE-*Vegfr2* sgRNA3 (referred to as WT-CjABE-sgRNA3) and LDE-CjABE-*Vegfr2* sgRNA3 (referred to as LDE-CjABE-sgRNA3) were used to test the A-to-G editing efficiency *in vivo* (Figure 3A). Subretinal injection was performed with 5×10^9 viral genomes per eye. Retinal pigment epithelial (RPE) cells from mice were collected to evaluate the editing efficiency 2 weeks or 6 weeks post-injection (Figure 4C).

Two weeks post-injection, both the retina and choroid exhibited prominent green fluorescence, confirming the successful infection of RPE cells (Figure S10A). No notable retinal editing could be observed, while the editing efficiency of WT-CjABE was 16% and that of LDE-CjABE was 23% in the choroid 2 weeks post infection (Figure S10B). To further evaluate the long-term performance and safety of LDE-CjABE, the mouse retina was isolated 6 weeks post-injection (Figure 4D), and PCR confirmed that AAV existed in each injected eye (Figure 4E). The A-to-G editing efficiency of WT-CjABE was 3.2%, and LDE-CjABE had an editing efficiency of 8.3% (Figure 4F). To investigate the specificity of LDE-CjABE, we used Cas-OFFinder to analyze the potential off-target sites of CjABE m*Vegfr2* sgRNA3 (Table S3). Through PCR and deep sequencing analysis, no off-target editing was detected at any of the eight putative

VEGFA3, and *HPD* target sites in HEK293T cells. (E) The A-to-G editing activity of WT-CjABE and LDE-CjABE across the editing window for 20 endogenous target sites shown in Table S5. Each nucleotide's position is numbered relative to the start of the target sequences. Control: GFP. Fold changes compared with wild-type CjCas9 are shown on the top of each bar. Data represent three biological repeats and are presented as mean ± SEM. Statistical significance was determined using one-way ANOVA (*$p < 0.05$, **$p < 0.01$, ***$p < 0.001$).

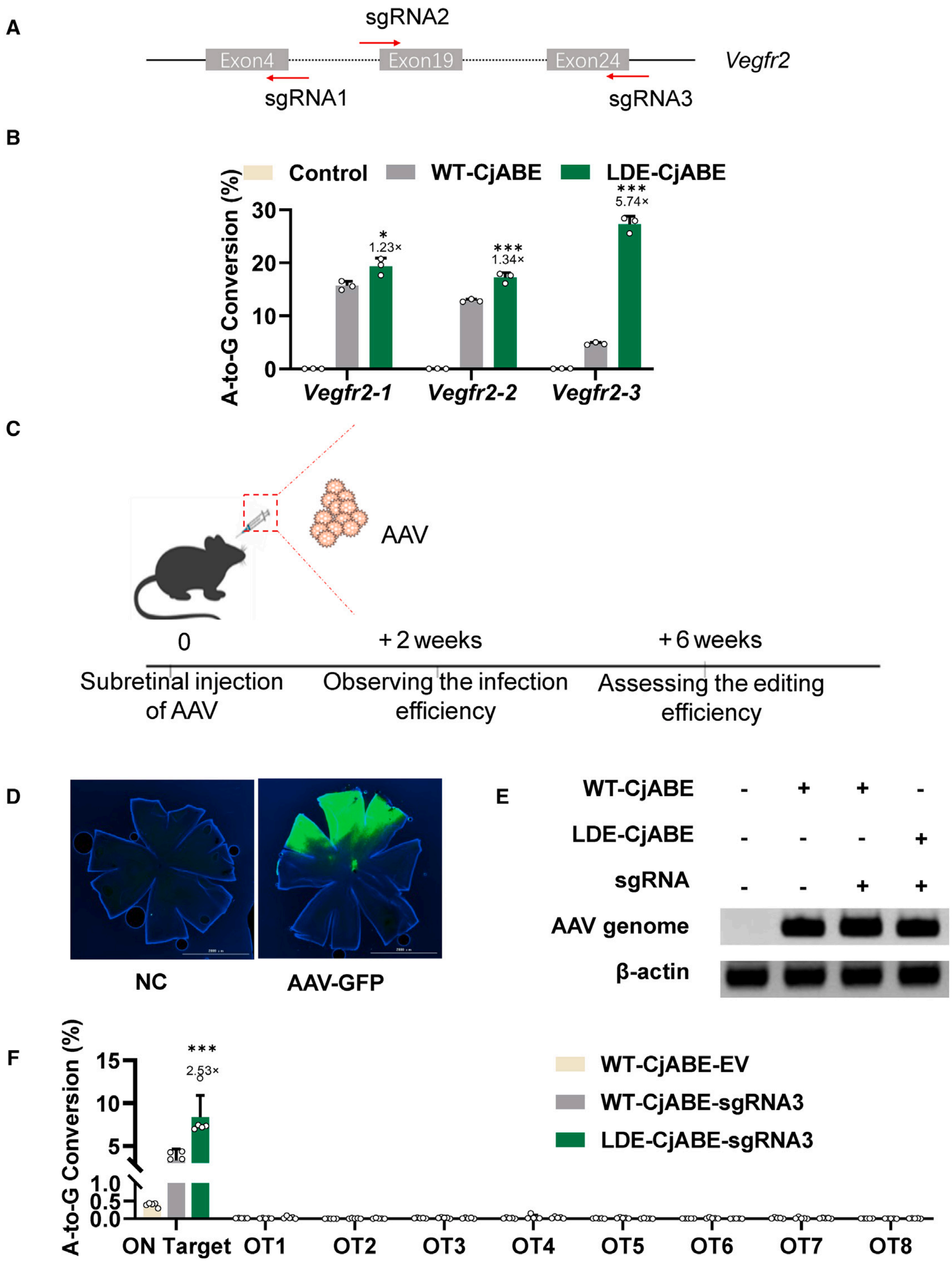

Figure 4. *In vivo* base editing with compact LDE-CjABE

(A) Schematic of CjCas9 sgRNAs targeting the mRNA splice sites of *Vegfr2*. Arrow directions indicate the 5′–3′ sequence of the spacer, with PAM in red and the target sequence in blue. (B) A-to-G conversion frequencies of CjABEs at the *Vegfr2* target sites in mouse Hepa1-6 cells. (C) Illustration of subretinal injection and experiment procedure. AAV was injected into the mouse retina, with genomic DNA isolated and extracted after 2 or 6 weeks. (D) Representative retinal flat-mount 6 weeks post-injection

(legend continued on next page)

off-target sites, demonstrating that LDE-CjABE was as specific as WT-CjABE (Figure 4F). Collectively, these data proved that LDE-CjABE delivered by an all-in-one AAV vector could edit target genes efficiently and precisely *in vivo*.

DISCUSSION

In this study, we demonstrated that enhancing the interaction between Cas9 and the target DNA backbone phosphate by replacing negatively charged (or neutral) residues with positively charged residues could boost the on-target binding capacity of CjCas9. This strategy can be applied to improve the performance of other Cas proteins. In addition, we used improved engineered CjCas9 variants (LDE-CjCas9) for gene transcription activation and base editing. The efficiency of LDE-dCjCas9-VPR was comparable to that of the dSpCas9-VPR system at the two sites. We found that LDE-CjABE and LDE-CjCBE exhibited higher editing efficiencies at multiple genomic loci (2- to 6-fold) than WT-CjABE and WT-CjCBE, respectively. Moreover, LDE-CjABE delivered by AAV vector showed efficient A-to-G editing without detectable off-target effects in the mouse retina.

Other approaches have been employed to increase the cleavage activity of CjCas9. enCjCas9, bearing the L58Y and D900K mutations to improve the binding of U40 to the sgRNA and the DNA backbone phosphate within the PAM motif, was engineered through structure-based mutagenesis and screened out through *in vitro* cleavage assay to increase its cleavage activity.[22] The broader PAM variant EvoCjCas9 (L58Y, E789K, N821K, and S951G), in which the mutations also stabilized sgRNA interaction and PAM recognition, was generated through phage-assisted continuous directed evolution (PACE).[23] Using error-prone PCR libraries, UltraCjCas9 (V35A, E189G, F214I, A492V, and T913S) with enhanced editing efficiency through neutralization of negative charge and modulation of PAM relaxation were screened out using the EPICA platform in yeast cells.[24] We compared enCjCas9, evoCjCas9, UltraCjCas9, and LDE-CjCas9 in transcriptional activation and base editing, and found diverse efficiencies among different targets (Figures 2 and 3). Upon our scope, in-depth investigation on the sequence preference of different engineered CjCas9s may help the researchers find the best variant for editing a specific target.[25] In our study, we mainly focused on the amino acids that may enhance the interaction between the DNA backbone phosphate and Cas protein (Figure 1A), and it may be possible to further enhance CjCas9 by combining the LDE mutation with enCjCas9, EvoCjCas9, and UltraCjCas9. Notably, some aspects should be considered for further engineering of CjCas9. Proximal CRISPR targeting may enhance the editing efficiency of CjCas9 target DNA cleavage, reflecting the limitation of target site accessibility.[30] Stabilizing CjCas9 by inhibiting proteasome and enhancing sgRNA stability could mildly enhance the efficiency at the FXN target site.[31] Guide-free CjCas9 may induce non-specific DNA damage in cells.[32] Taken together, these facts limit the application of CjCas9, and in addition to the binding and cleavage activities, further optimization of CjCas9 could focus more on target site accessibility and cytotoxicity.

For CjABEs, we observed editing on A1-A17 within the sgRNA protospacer, which is wider than the BEs based on SpCas9. This may be due to the smaller steric hindrance of CjCas9, allowing the deaminase to approach more bases on the non-target strand. Similar to CjCas9 BEs, SaCas9 BEs also have a broader editing window than SpCas9 BEs.[33,34] The characteristic of a broad active window might be beneficial for gene inactivation and long-range mutantgenesis.[35] Although the broad active window may lead to bystander editing of other As within the editing window when targeting specific loci, it did not affect the disruption of *Vegfr*2 mRNA splicing in this study. However, this feature severely limits the application of CjABE in precise editing therapies, making it difficult to precisely repair single-base mutations at disease loci. To narrow the editing window of BEs based on CjCas9, engineered deaminases with a narrowed editing window (such as TadA-9) could be utilized.[36] Additionally, linker length optimization could be performed, which may narrow the activity window of CjABE. In addition, we fused a smaller cytidine deaminase with LDE-CjCas9 nickase to develop CjCBE, but its editing efficiency was low. In addition to the LDE-CjCBE developed in our study, recent studies reported that CjCas9 nickase fused with PmCDA1 or APOBEC1 could effectively induce C-to-T conversions.[22,37] However, neither of these cytosine deaminase fusions could meet the requirement to be packaged in an all-in-one AAV vector (<4.7 kb). Recently, David R. Liu reported using phage-assisted continuous evolution to obtain TadA-derived cytosine BEs (TadCBE).[38] Fusing the deaminase of TadCBE to LDE-CjCas9 nickase may increase C-to-T editing efficiency, which requires further study.

Recently, other compact CRISPR systems (e.g., Cas12f) have been developed.[39] Although Cas12f is smaller, the study by Lukas Schmidheini et al. showed that the genome-editing capabilities of engineered Cas12f (CasMINI) were comparable to CjCas9.[23] Compared with the type V Cas proteins, which have only the RuvC domain, miniature Cas9s, such as CjCas9, have both RuvC and HNH domains, giving the possibility to be engineered into nickase for high-efficiency base editing and prime editors employing endogenous DNA repair mechanism.[22] In addition, the PAM-modified CjCas9 offers an alternative targeting range (PAM: N4AH and N5HA),[22,23] which complements the targeting range of Cas12f (PAM: TTR). In combination with the PAM-modified CjCas9, LDE-CjABE and LDE-CjCBE may have a border target scope for base editing. In conclusion, these compact Cas proteins were designed to broaden the applications of BEs in biomedical research.

of AAV-GFP, the mouse retina was isolated. Scale bar, 2,000 μm. (E) PCR amplification detecting the AAV genome in mouse retina post-injection with WT-CjABE-EV, WT-CjABE-sgRNA3, or LDE-CjABE-sgRNA3. NC: AAV-GFP. (F) A-to-G editing efficiencies of AAV-CjABE at the *Vegfr2-3* site and eight predicted off-target sites of *Vegfr2-3* in mouse retina. Fold changes compared with wild-type CjCas9 are shown on the top of each bar. Data represent three (B) or five (F) biological repeats and are presented as mean ± SEM. Statistical significance was determined using one-way ANOVA (*$p < 0.05$, **$p < 0.01$, ***$p < 0.001$).

MATERIALS AND METHODS

Vector construction

The full-length coding sequences of CjCas9 were synthesized by IGE Biotechnology (Guangzhou, China). Mutations were introduced into plasmids by overlap PCR. Oligos containing spacer sequences were annealed and ligated into puc19-CjCas9-sgRNA for sgRNA expression in mammalian cells. sgRNA targets are listed in Tables S4–S6. dCjCas9 contains the mutations D8A and H559A, while nCjCas9 contains the mutation D8A.

Animals

C57BL/6 mice were purchased from SPF Biotechnology (Beijing, China) and housed under standard conditions (22 ± 1°C) in a specific pathogen-free animal facility with a 14/10-h light-dark cycle at Sun Yat-sen University. The Institutional Animal Care and Use Committee of Sun Yat-sen University, P.R. China, approved all animal experiments.

Cell culture

HEK293T and Hepa1-6 cells were obtained from ATCC, all of which were cultured in DMEM (Corning, 10-013-CV) supplemented with 10% FBS (Lonsera, S711-001S) at 37°C with 5% CO_2. For sgRNA targeting validation, HEK293T cells were seeded onto a 12-well plate at a density of 2×10^5 cells/well on the day before transfection. Transient transfection of HEK293T cells was performed using polyethylenimine PEI (Polysciences, 24765–1). A total of 1,500 ng DNA was seeded into each well of a 12-well plate. Cells were collected 3 days post-transfection for further analysis. The GFP reporter cell line was constructed by infecting HEK293T cells with a lentiviral packaging of the CMVmini promoter and GFP element.

Flow cytometry analysis

The cells were digested with 0.25% trypsin, and trypsin digestion was terminated using DMEM containing 10% FBS. Cells were collected and suspended in phosphate-buffered saline (PBS). GFP-positive cells were detected using CytoFLEX (Beckman Coulter).

Western blot

Cultured cells were lysed in radioimmunoprecipitation assay (RIPA) buffer (GBCBIO, G3424) containing a protease inhibitor cocktail (Beyotime, P1005). Samples were centrifuged at 14,000 $\times$ g for 10 min. The supernatant was harvested and quantified using a BCA protein assay kit (Thermo Fisher, 23225) on Victor X5. Twenty-five micrograms of protein was mixed and boiled with 5 $\times$ SDS loading buffer. The samples were then subjected to 10% SDS-PAGE and transferred to nitrocellulose membranes for 1 h in a transfer buffer at 300 mA using a Trans-Blot Turbo Transfer System (Bio-Rad, USA). The blots were then blocked with 5% bovine serum albumin (Solarbio, A8010) for 1 h and incubated overnight at 4°C with primary antibodies. The blots were washed with Tris-buffered saline containing 0.1% Tween 20 before incubation with a secondary antibody for 2 h. Bands were detected and imaged using the Odyssey system (LI-COR, USA), and band intensity was measured using ImageJ software (NIH image, USA).

DNA isolation from cells

Seventy-two hours after transfection, the cells were washed twice with PBS before extraction using the AxyPrep Blood Genomic DNA Mini-prep Kit (Axygen, AP-MN-BL-GDNA-250). Cell genomic DNA was then used as a template for polymerase chain reaction amplification. Amplicons were subjected to Sanger sequencing and analyzed by tracking of indels by decomposition (TIDE)24 or EditR.

Quantitative PCR

Briefly, total RNA was extracted using TRIZOL (Thermo Fisher, 9109) following the manufacturer's instructions and quantified using a Nanodrop 1000 (Thermo Fisher, USA). Reverse transcription was performed using the PrimeScriptRT Reagent Kit (TAKARA, RR047Q) following the manufacturer's instructions. Quantitative PCR was carried out in the qTOWER3 system (Analytikjena, DE) using TAKARA TB Green II Real-Time PCR Master Mix, according to the manufacturer's instructions. Quantitative PCR was performed using the indicated primers for specific genes, and GAPDH served as a control. Relative expression levels were determined using the $-\Delta\Delta Ct$ method. Primers used for qPCR are listed in Table S7.

Deep sequencing sample preparation

Cell genomic DNA was then used as a template for PCR amplification. Target sites were amplified by PCR using barcoded primers and KOD FX (Toyobo, KFX-101). Before deep sequencing, the PCR product was purified using the QIAquick PCR Purification Kit (QIAGEN, 28106). Deep sequencing was performed by Novogene (Beijing, China) using a paired-end 150-bp Hiseq 2500 platform (Illumina, USA). The primers used to generate the amplicons are listed in Table S7. Finally, the data were determined using MATLAB script, as previously reported.[5]

AAV production

For AAV production, HEK293T cells were plated onto 15-cm dishes at a density of 1×10^7 cells/dish. After 24 h, the cells were transfected with PEI. Briefly, for each 15-cm dish, 12 μg transfer plasmids and 20 μg pAAV8 (MiaoLing Plasmid Sharing Platform, P13271) were diluted in 1 mL Opti-MEM (GIBCO, 31985070). Next, 96 μg of PEI diluted in 1 mL Opti-MEM was mixed with the diluted plasmids. The mixture was incubated at room temperature for 15 min before being added to the cultured cells. AAV was collected 96 h post-transfection, according to the protocol provided by the Addgene website (https://www.addgene.org/protocols/aav-production-hek293-cells/). Briefly, the cell culture medium was collected and subjected to polyethylene glycol (PEG) 8000 (Sigma-Aldrich, P5413) precipitation. The detached cells were lysed by sonication to release AAV particles. Then, the collected crude AAV solution was purified and concentrated by iodixanol gradient ultracentrifugation and ultrafiltration according to the protocol provided by Addgene's website (https://www.addgene.org/protocols/aav-purification-iodixanol-gradient-ultracentrifugation/). Titers of AAV stocks were determined by qRT-PCR using TB Green (Takara, RR430). Before releasing the viral DNA from the particles, all extra-viral DNA was removed by digestion with recombinant DNase I (Takara, 2270A). The viral DNA was released by incubation

with proteinase K (Takara, 9034) for 60 min at 50°C, followed by 10 min of inactivation at 90°C. The primers targeting AAV ITR (forward primer [FP]: 5′-GGAACCCCTAGTGATGGAGTT-3′; reverse primer [RP]: 5′-CGGCCTCAGTGAGCGA-3′) were used.[40]

Subretinal injection

Six-week-old C57 BL/6 male mice were anesthetized. Phenylephrine (0.5%) and tropicamide (0.5%) were used to dilate pupils. Proparacaine hydrochloride (0.5%) was then used to anesthetize the eyes. For the assisted visualization of the mouse retina, a small drop of ophthalmic viscoelastic solution was placed on the corneal surface. A small hole was then punctured at the corneal margin using a 30G needle. One microliter of AAV8 (5×10^9 vg) was injected using a 33G blunt-end needle (Hamilton; 7803–05) in 40 s, which was connected to a Hamilton syringe (1701RN no needle, 7653–01) and inserted through the hole and positioned in the subretinal space. To prevent dryness and post-surgical infection, vet ointment was used post-injection.

Mouse retinal processing

Two weeks post-injection, for mouse retinal flat-mounts, the mouse retina was isolated and cut to a "clover" shape to release any tensions in the PBS. The isolated retina was stained in 0.1% Hoechst and mounted in 7.5% polyvinylpyrrolidone, 50 mM Tris (pH 8.0), mounting media. Six weeks after infection, the retina was isolated to detect editing efficiency. RPE cells were collected from the choroid after digestion with 0.25% trypsin at 37°C for 1 h and shaking for 20 min. DNA extraction was later performed to detect editing efficiency.

In silico off-target prediction

Cas-OFFinder was used to predict the off-target binding sites of CjCas9 sgRNA in the mouse (*Mus musculus* [mm10]) reference genomes with the following parameters: Pam type = CjCas9 (5′-NNNNRYAC-3′), mismatch number = 4, DNA bulge size = 0, and RNA bulge size = 0. Primers were designed to amplify the loci flanking the top eight matches and are listed in Table S6.

Protein expression and purification

His-tagged recombinant proteins were purified as previously reported with minor modifications.[41] Briefly, BL21 StarTM (DE3) *E. coli* cells (Thermo Fisher, USA) transformed with pET28a-DE-CjCas9-EGFP, pET28a-LDE-CjCas9-EGFP, or pET28a-CjCas9-EGFP were cultured overnight until OD_{600} reached 0.5–0.6 before the addition of IPTG (0.5 mM) and induction at 18 °C for 14–16 h. Cells were lysed in lysis buffer (100 mM Tris-HCl, pH 8.0, 1 M NaCl, 20% glycerol, 5 mM tris(2-carboxyethyl)phosphine (TCEP; Sigma-Aldrich, DE), 20 mM imidazole (Sigma-Aldrich, DE), and protease inhibitors), followed by sonication. The supernatant was then incubated with Ni-NTA agarose resin (GE Healthcare) and washed in wash buffer (100 mM Tris-HCl, pH 8.0, 0.5 M NaCl, 20% glycerol, 5 mM TCEP, and 20 mM imidazole) before elution (100 mM Tris-HCl, pH 8.0, 0.5 M NaCl, 20% glycerol, 5 mM TCEP, and 270 mM imidazole). All proteins were further purified on a 5-mL Hi-Trap HP SP cation exchange column (GE Healthcare, UK), concentrated with a Microcon-30 kDa Centrifugal Filter Unit (30 kDa cutoff) (EMD Millipore, USA), sterile filtered (0.22 μm PVDF membrane) (EMD Millipore, USA), and quantified using the Reducing Agent Compatible Bicinchoninic acid assay (Pierce Biotechnology). The purified proteins were aliquoted and snap-frozen in liquid nitrogen for storage at −80°C.

Microscale thermophoresis

The MST methodology was performed in accordance with previous articles.[42] WT dCjCas9-EGFP and LDE-dCjCas9-EGFP were purified using an *in vitro* prokaryotic system. sgRNAs were synthesized through *in vitro* transcription and dsDNA was generated by annealing complementary DNA strands. The binding ability of dsDNA or sgRNA to various CjCas9 mutants was quantified using a Monolith NT.115 instrument (NanoTemper Technologies, Germany). The dCjCas9-EGFP fusion protein or the dCjCas9-EGFP/gRNA RNP complex (protein: gRNA = 1:1) was diluted with binding buffer (50 mM Tris-HCl, pH 7.4, 150 mM NaCl, 0.05% Tween 20, and 10 mM MgCl2) to obtain a final concentration of 50 nM. Equivolume mixing of the protein with double-stranded DNA (dsDNA) or sgRNA, ranging from 0.305 nM to 2,500 nM for dsDNA and 0.061 nM–2,000 nM for sgRNA, was conducted. After a 10-min incubation at room temperature, the samples were loaded into NanoTemper standard capillaries for analysis at 25°C, using high MST and 40% LED power settings. Data were processed using NanoTemper's MO affinity analysis software and subsequently graphed using Origin 2024 (OriginLab, Northampton, MA, USA).

Statistical analysis

All data are expressed as mean ± SEM of at least three replicates for all experiments. One-way ANOVA was performed using GraphPad Prism software v8.0.1 to compare the editing efficiencies of different variants. The probability value < 0.05 ($p < 0.05$) indicates statistical significance. n.s., not significant, *$p < 0.05$, **$p < 0.01$, ***$p < 0.001$.

DATA AND CODE AVAILABILITY

Data that support the findings of this study are available from the corresponding author upon reasonable request.

ACKNOWLEDGMENTS

We would like to thank all the members of our lab for their helpful discussions. This work was supported by the National Key R&D Program of China (2023YFC3403400), the National Natural Science Foundation of China (32471506, 32371509, 32001063), the Guangdong Special Support Program (2019BT02Y276), the Guangdong Basic and Applied Basic Research Foundation (2023A1515010176), the Guangzhou Science and Technology Planning Project (2023A04J1952), and the Fundamental Research Funds for the Central Universities, Sun Yat-sen University (23ptpy59).

AUTHOR CONTRIBUTIONS

Y.C. and P.L. designed the experiments. Y.C., R.K., Q.Z., Y.Y., J.L., Guanglan Wu, Q.L., S.H., X.L., Guifang Wu, and K.C. performed the experiments. P.L. supervised the research. All authors discussed the results and commented on the manuscript.

DECLARATION OF INTERESTS

The authors declare no competing interests.

SUPPLEMENTAL INFORMATION

Supplemental information can be found online at https://doi.org/10.1016/j.omtn.2024.102366.

REFERENCES

1. Anzalone, A.V., Koblan, L.W., and Liu, D.R. (2020). Genome editing with CRISPR-Cas nucleases, base editors, transposases and prime editors. Nat. Biotechnol. *38*, 824–844. https://doi.org/10.1038/s41587-020-0561-9.

2. Fellmann, C., Gowen, B.G., Lin, P.C., Doudna, J.A., and Corn, J.E. (2017). Cornerstones of CRISPR-Cas in drug discovery and therapy. Nat. Rev. Drug Discov. *16*, 89–100. https://doi.org/10.1038/nrd.2016.238.

3. Rees, H.A., and Liu, D.R. (2018). Base editing: precision chemistry on the genome and transcriptome of living cells. Nat. Rev. Genet. *19*, 770–788. https://doi.org/10.1038/s41576-018-0059-1.

4. Komor, A.C., Kim, Y.B., Packer, M.S., Zuris, J.A., and Liu, D.R. (2016). Programmable editing of a target base in genomic DNA without double-stranded DNA cleavage. Nature *533*, 420–424. https://doi.org/10.1038/nature17946.

5. Gaudelli, N.M., Komor, A.C., Rees, H.A., Packer, M.S., Badran, A.H., Bryson, D.I., and Liu, D.R. (2017). Programmable base editing of A.T to G.C in genomic DNA without DNA cleavage. Nature *551*, 464–471. https://doi.org/10.1038/nature24644.

6. Xu, L., Zhang, C., Li, H., Wang, P., Gao, Y., Mokadam, N.A., Ma, J., Arnold, W.D., and Han, R. (2021). Efficient precise in vivo base editing in adult dystrophic mice. Nat. Commun. *12*, 3719. https://doi.org/10.1038/s41467-021-23996-y.

7. Chen, Y., Zhi, S., Liu, W., Wen, J., Hu, S., Cao, T., Sun, H., Li, Y., Huang, L., Liu, Y., et al. (2020). Development of Highly Efficient Dual-AAV Split Adenosine Base Editor for In Vivo Gene Therapy. Small Methods *4*, 2000309. https://doi.org/10.1002/smtd.202000309.

8. Hu, Z., Wang, S., Zhang, C., Gao, N., Li, M., Wang, D., Wang, D., Liu, D., Liu, H., Ong, S.G., et al. (2020). A compact Cas9 ortholog from Staphylococcus Auricularis (SauriCas9) expands the DNA targeting scope. PLoS Biol. *18*, e3000686. https://doi.org/10.1371/journal.pbio.3000686.

9. Chen, S., Liu, Z., Xie, W., Yu, H., Lai, L., and Li, Z. (2022). Compact Cje3Cas9 for Efficient In Vivo Genome Editing and Adenine Base Editing. CRISPR J. *5*, 472–486. https://doi.org/10.1089/crispr.2021.0143.

10. Chang, Y.J., Bae, J., Zhao, Y., Lee, G., Han, J., Lee, Y.H., Koo, O.J., Seo, S., Choi, Y.K., and Yeom, S.C. (2020). In vivo multiplex gene targeting with Streptococcus pyogens and Campylobacter jejuni Cas9 for pancreatic cancer modeling in wild-type animal. J. Vet. Sci. *21*, e26. https://doi.org/10.4142/jvs.2020.21.e26.

11. Kim, E., Koo, T., Park, S.W., Kim, D., Kim, K., Cho, H.Y., Song, D.W., Lee, K.J., Jung, M.H., Kim, S., et al. (2017). In vivo genome editing with a small Cas9 orthologue derived from Campylobacter jejuni. Nat. Commun. *8*, 14500. https://doi.org/10.1038/ncomms14500.

12. Yamada, M., Watanabe, Y., Gootenberg, J.S., Hirano, H., Ran, F.A., Nakane, T., Ishitani, R., Zhang, F., Nishimasu, H., and Nureki, O. (2017). Crystal Structure of the Minimal Cas9 from Campylobacter jejuni Reveals the Molecular Diversity in the CRISPR-Cas9 Systems. Mol. Cell *65*, 1109–1121.e3. https://doi.org/10.1016/j.molcel.2017.02.007.

13. Jo, D.H., Koo, T., Cho, C.S., Kim, J.H., Kim, J.S., and Kim, J.H. (2019). Long-Term Effects of In Vivo Genome Editing in the Mouse Retina Using Campylobacter jejuni Cas9 Expressed via Adeno-Associated Virus. Mol. Ther. *27*, 130–136. https://doi.org/10.1016/j.ymthe.2018.10.009.

14. Li, F., Wing, K., Wang, J.H., Luu, C.D., Bender, J.A., Chen, J., Wang, Q., Lu, Q., Nguyen Tran, M.T., Young, K.M., et al. (2020). Comparison of CRISPR/Cas Endonucleases for in vivo Retinal Gene Editing. Front. Cell. Neurosci. *14*, 570917. https://doi.org/10.3389/fncel.2020.570917.

15. Ma, E., Harrington, L.B., O'Connell, M.R., Zhou, K., and Doudna, J.A. (2015). Single-Stranded DNA Cleavage by Divergent CRISPR-Cas9 Enzymes. Mol. Cell *60*, 398–407. https://doi.org/10.1016/j.molcel.2015.10.030.

16. Slaymaker, I.M., Gao, L., Zetsche, B., Scott, D.A., Yan, W.X., and Zhang, F. (2016). Rationally engineered Cas9 nucleases with improved specificity. Science *351*, 84–88.

17. Kleinstiver, B.P., Pattanayak, V., Prew, M.S., Tsai, S.Q., Nguyen, N.T., Zheng, Z., and Joung, J.K. (2016). High-fidelity CRISPR-Cas9 nucleases with no detectable genome-wide off-target effects. Nature *529*, 490–495. https://doi.org/10.1038/nature16526.

18. Tan, Y., Chu, A.H.Y., Bao, S., Hoang, D.A., Kebede, F.T., Xiong, W., Ji, M., Shi, J., and Zheng, Z. (2019). Rationally engineered Staphylococcus aureus Cas9 nucleases with high genome-wide specificity. Proc. Natl. Acad. Sci. USA *116*, 20969–20976. https://doi.org/10.1073/pnas.1906843116.

19. Marcovitz, A., and Levy, Y. (2011). Frustration in protein-DNA binding influences conformational switching and target search kinetics. Proc. Natl. Acad. Sci. USA *108*, 17957–17962. https://doi.org/10.1073/pnas.1109594108.

20. Chen, Y., Liu, J., Zhi, S., Zheng, Q., Ma, W., Huang, J., Liu, Y., Liu, D., Liang, P., and Songyang, Z. (2020). Repurposing type I-F CRISPR-Cas system as a transcriptional activation tool in human cells. Nat. Commun. *11*, 3136. https://doi.org/10.1038/s41467-020-16880-8.

21. Liu, Q., Chen, Y., Hu, S., Liu, W., Xie, D., Yang, X., Huang, W., Liu, S., Chen, X., Liu, H., and Huang, J. (2023). Screening an Effective Dual-Adeno-Associated Virus Split-Cytosine Base Editor System for C-to-T Conversion. Hum. Gene Ther. *34*, 629–638. https://doi.org/10.1089/hum.2023.055.

22. Nakagawa, R., Ishiguro, S., Okazaki, S., Mori, H., Tanaka, M., Aburatani, H., Yachie, N., Nishimasu, H., and Nureki, O. (2022). Engineered Campylobacter jejuni Cas9 variant with enhanced activity and broader targeting range. Commun. Biol. *5*, 211. https://doi.org/10.1038/s42003-022-03149-7.

23. Schmidheini, L., Mathis, N., Marquart, K.F., Rothgangl, T., Kissling, L., Böck, D., Chanez, C., Wang, J.P., Jinek, M., and Schwank, G. (2024). Continuous directed evolution of a compact CjCas9 variant with broad PAM compatibility. Nat. Chem. Biol. *20*, 333–343. https://doi.org/10.1038/s41589-023-01427-x.

24. Ruta, G.V., Ciciani, M., Kheir, E., Gentile, M.D., Amistadi, S., Casini, A., and Cereseto, A. (2024). Eukaryotic-driven directed evolution of Cas9 nucleases. Genome Biol. *25*, 79. https://doi.org/10.1186/s13059-024-03215-9.

25. Li, J., Wu, P., Cao, Z., Huang, G., Lu, Z., Yan, J., Zhang, H., Zhou, Y., Liu, R., Chen, H., et al. (2024). Machine learning-based prediction models to guide the selection of Cas9 variants for efficient gene editing. Cell Rep. *43*, 113765. https://doi.org/10.1016/j.celrep.2024.113765.

26. Hand, T.H., Roth, M.O., Smith, C.L., Shiel, E., Klein, K.N., Gilbert, D.M., and Li, H. (2021). Catalytically Enhanced Cas9 through Directed Protein Evolution. CRISPR J. *4*, 223–232. https://doi.org/10.1089/crispr.2020.0092.

27. Das, A., Hand, T.H., Smith, C.L., Wickline, E., Zawrotny, M., and Li, H. (2020). The molecular basis for recognition of 5'-NNNCC-3' PAM and its methylation state by Acidothermus cellulolyticus Cas9. Nat. Commun. *11*, 6346. https://doi.org/10.1038/s41467-020-20204-1.

28. Legut, M., Daniloski, Z., Xue, X., McKenzie, D., Guo, X., Wessels, H.H., and Sanjana, N.E. (2020). High-Throughput Screens of PAM-Flexible Cas9 Variants for Gene Knockout and Transcriptional Modulation. Cell Rep. *30*, 2859–2868.e5. https://doi.org/10.1016/j.celrep.2020.02.010.

29. Thuronyi, B.W., Koblan, L.W., Levy, J.M., Yeh, W.H., Zheng, C., Newby, G.A., Wilson, C., Bhaumik, M., Shubina-Oleinik, O., Holt, J.R., and Liu, D.R. (2019). Continuous evolution of base editors with expanded target compatibility and improved activity. Nat. Biotechnol. *37*, 1070–1079. https://doi.org/10.1038/s41587-019-0193-0.

30. Chen, F., Ding, X., Feng, Y., Seebeck, T., Jiang, Y., and Davis, G.D. (2017). Targeted activation of diverse CRISPR-Cas systems for mammalian genome editing via proximal CRISPR targeting. Nat. Commun. *8*, 14958. https://doi.org/10.1038/ncomms14958.

31. Yameogo, P., Majeau, N., Mbakam, C.H., and Tremblay, J.P. (2023). Small-molecule inhibitors of proteasome increase CjCas9 protein stability. PLoS One *18*, e0280353. https://doi.org/10.1371/journal.pone.0280353.

32. Saha, C., Mohanraju, P., Stubbs, A., Dugar, G., Hoogstrate, Y., Kremers, G.J., van Cappellen, W.A., Horst-Kreft, D., Laffeber, C., Lebbink, J.H.G., et al. (2020). Guide-free Cas9 from pathogenic Campylobacter jejuni bacteria causes severe damage to DNA. Sci. Adv. *6*, eaaz4849. https://doi.org/10.1126/sciadv.aaz4849.

33. Kim, Y.B., Komor, A.C., Levy, J.M., Packer, M.S., Zhao, K.T., and Liu, D.R. (2017). Increasing the genome-targeting scope and precision of base editing with engineered

Cas9-cytidine deaminase fusions. Nat. Biotechnol. *35*, 371–376. https://doi.org/10.1038/nbt.3803.

34. Huang, T.P., Zhao, K.T., Miller, S.M., Gaudelli, N.M., Oakes, B.L., Fellmann, C., Savage, D.F., and Liu, D.R. (2019). Circularly permuted and PAM-modified Cas9 variants broaden the targeting scope of base editors. Nat. Biotechnol. *37*, 626–631. https://doi.org/10.1038/s41587-019-0134-y.
35. Rothgangl, T., Dennis, M.K., Lin, P.J.C., Oka, R., Witzigmann, D., Villiger, L., Qi, W., Hruzova, M., Kissling, L., Lenggenhager, D., et al. (2021). In vivo adenine base editing of PCSK9 in macaques reduces LDL cholesterol levels. Nat. Biotechnol. *39*, 949–957. https://doi.org/10.1038/s41587-021-00933-4.
36. Chen, L., Zhang, S., Xue, N., Hong, M., Zhang, X., Zhang, D., Yang, J., Bai, S., Huang, Y., Meng, H., et al. (2023). Engineering a precise adenine base editor with minimal bystander editing. Nat. Chem. Biol. *19*, 101–110. https://doi.org/10.1038/s41589-022-01163-8.
37. Kweon, J., Jang, A.H., Kwon, E., Kim, U., Shin, H.R., See, J., Jang, G., Lee, C., Koo, T., Kim, S., and Kim, Y. (2023). Targeted dual base editing with Campylobacter jejuni Cas9 by single AAV-mediated delivery. Exp. Mol. Med. *55*, 377–384. https://doi.org/10.1038/s12276-023-00938-w.
38. Neugebauer, M.E., Hsu, A., Arbab, M., Krasnow, N.A., McElroy, A.N., Pandey, S., Doman, J.L., Huang, T.P., Raguram, A., Banskota, S., et al. (2023). Evolution of an adenine base editor into a small, efficient cytosine base editor with low off-target activity. Nat. Biotechnol. *41*, 673–685. https://doi.org/10.1038/s41587-022-01533-6.
39. Harrington, L.B., Burstein, D., Chen, J.S., Paez-Espino, D., Ma, E., Witte, I.P., Cofsky, J.C., Kyrpides, N.C., Banfield, J.F., and Doudna, J.A. (2018). Programmed DNA destruction by miniature CRISPR-Cas14 enzymes. Science *362*, 839–842. https://doi.org/10.1126/science.aav4294.
40. Aurnhammer, C., Haase, M., Muether, N., Hausl, M., Rauschhuber, C., Huber, I., Nitschko, H., Busch, U., Sing, A., Ehrhardt, A., and Baiker, A. (2012). Universal real-time PCR for the detection and quantification of adeno-associated virus serotype 2-derived inverted terminal repeat sequences. Hum. Gene Ther. Methods *23*, 18–28. https://doi.org/10.1089/hgtb.2011.034.
41. Liang, P., Xie, X., Zhi, S., Sun, H., Zhang, X., Chen, Y., Chen, Y., Xiong, Y., Ma, W., Liu, D., et al. (2019). Genome-wide profiling of adenine base editor specificity by EndoV-seq. Nat. Commun. *10*, 67. https://doi.org/10.1038/s41467-018-07988-z.
42. Acharya, S., Mishra, A., Paul, D., Ansari, A.H., Azhar, M., Kumar, M., Rauthan, R., Sharma, N., Aich, M., Sinha, D., et al. (2019). Francisella novicida Cas9 interrogates genomic DNA with very high specificity and can be used for mammalian genome editing. Proc. Natl. Acad. Sci. USA *116*, 20959–20968. https://doi.org/10.1073/pnas.1818461116.

OMTN, Volume *35*

Supplemental information

Rationally designed *Campylobacter jejuni* Cas9 enables efficient gene activation and base editing

Yuxi Chen, Rui Kang, Yuanling Jiang, Qi Zheng, Yue Yang, Jiaqi Liu, Guanglan Wu, Weijun Zhao, Zhan Li, Chengxiang Peng, Pengfei Zhang, Fei Peng, Qianyi Liu, Sihui Hu, Xiao Luo, Guifang Wu, Kaixin Cui, Junjiu Huang, Yongming Wang, Zhou Songyang, and Puping Liang

Figure S1

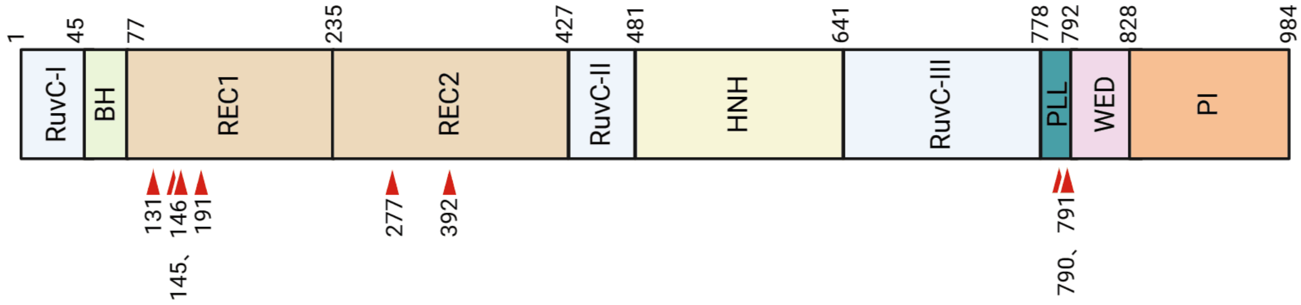

Figure S1: Schematic Overview of Mutations in CjCas9. This schematic provides a detailed view of the mutations introduced in the CjCas9 protein. The numbers correspond to the specific amino acid positions within the protein's structural domains. The red triangles denote the clarity locations where point mutations have been introduced within these domains.

Figure S2

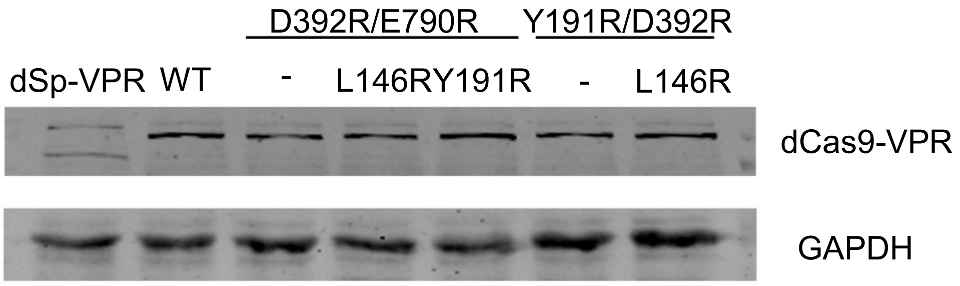

Figure S2. Western blot analysis of CjCas9 variant protein levels in HEK293T cells.

The lanes are arranged from left to right as follows: SpCas9-VPR, WT, D392R/E790R, D392R/E790R/L146R, D392R/E790R/Y191R, Y191R/D392R, and Y191R/D392R/L146R CjCas9-VPR.

Figure S3

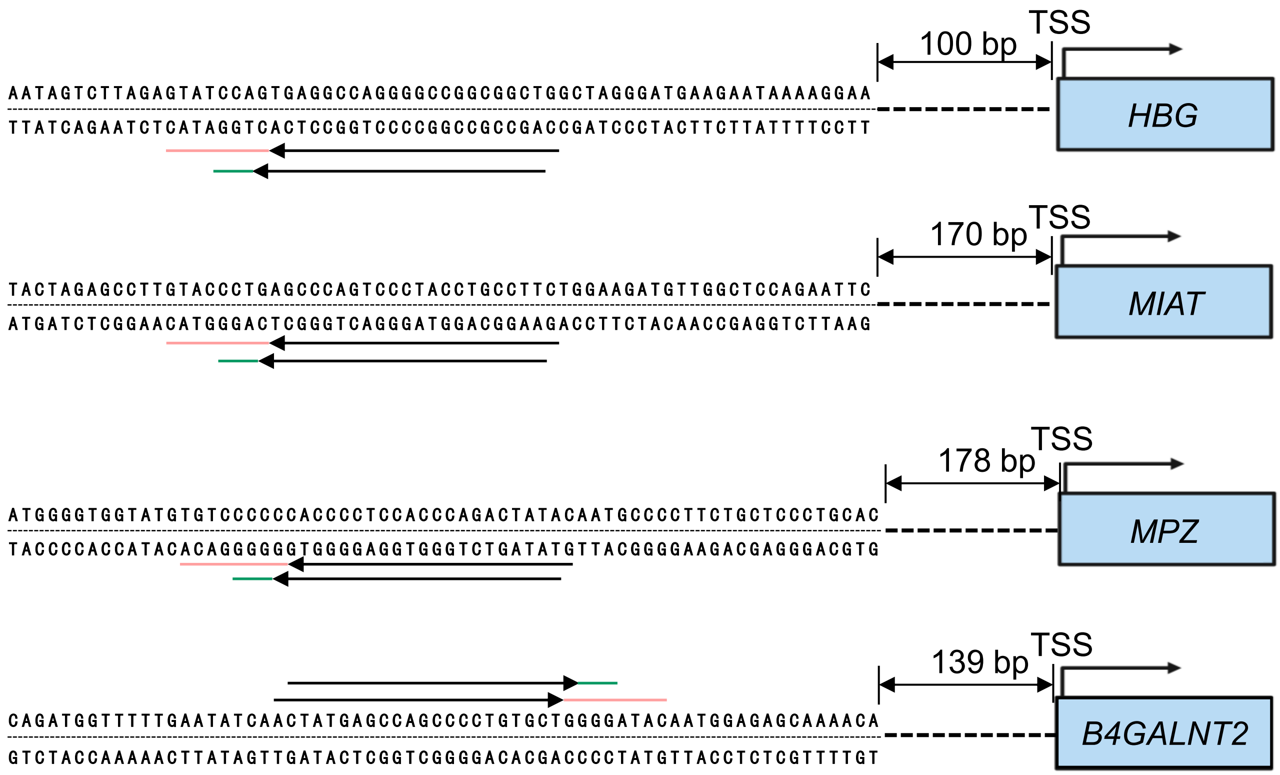

Figure S3. A schematic illustrating the sgRNAs of CjCas9 variants and SauriCas9 targeting regions upstream of the TSS for the *B4GALNT2, MIAT, HBG,* and *MPZ*. The orientation of the arrows signifies the direction of the 5′ to 3′ sequence of the spacer within the sgRNAs. The PAM motifs are distinguished by color-coded lines, with the CjCas9 variant depicted in pink and SauriCas9 in green referring to Figure 2C. TSS: transcription start site.

Figure S4

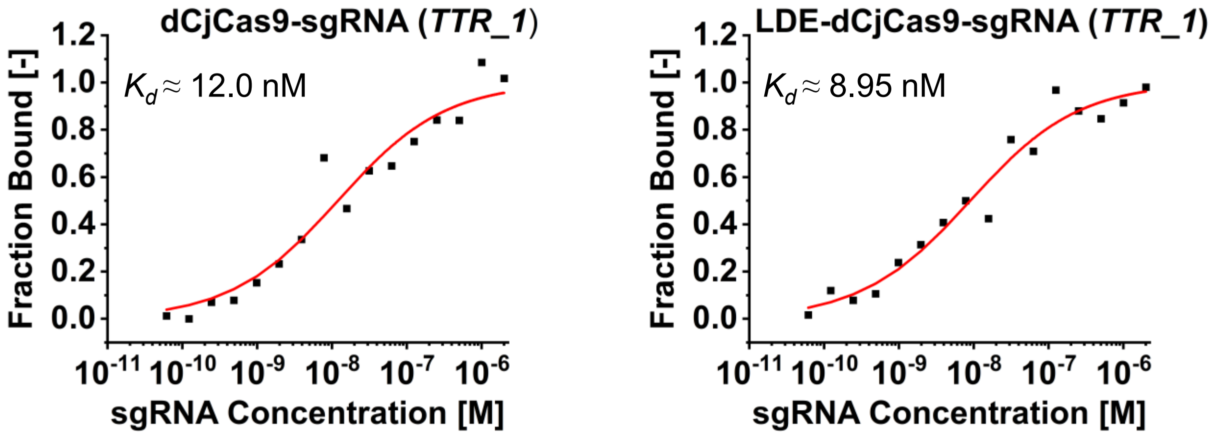

Figure S4. Microscale thermophoresis (MST) analysis of dCjCas9 or LDE-dCjCas9 protein binding to TTR sgRNA. The dissociation constants (Kd values), indicative of the strength of the protein-sgRNA interaction, were determined to be 12.0 nanomolar (nM) for dCjCas9 and 8.95 nanomolar (nM) for the LDE-dCjCas9 variant, respectively.

Figure S5

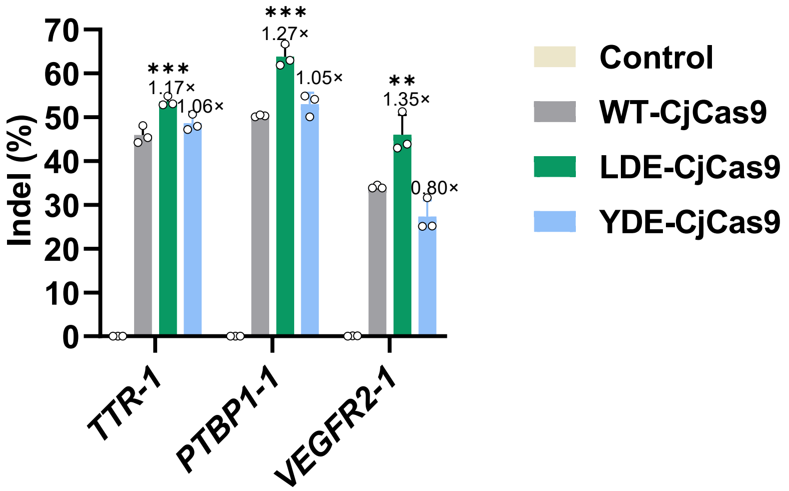

Figure S5. The indel frequencies induced by WT-CjCas9, LDE-CjCas9, and YDE-CjCas9 at the endogenous *TTR-1, PTBP1-1,* and *VEGFR2-1* target sites in HEK293T cells. Control: GFP. Fold changes comparing to wild type CjCas9 were shown on the top of each bar. Data represented three biological repeats and presented as mean ± S.E.M. Statistical significance was determined using two-tailed t test (*$P < 0.05$, **$P < 0.01$, ***$P < 0.001$)

Figure S6

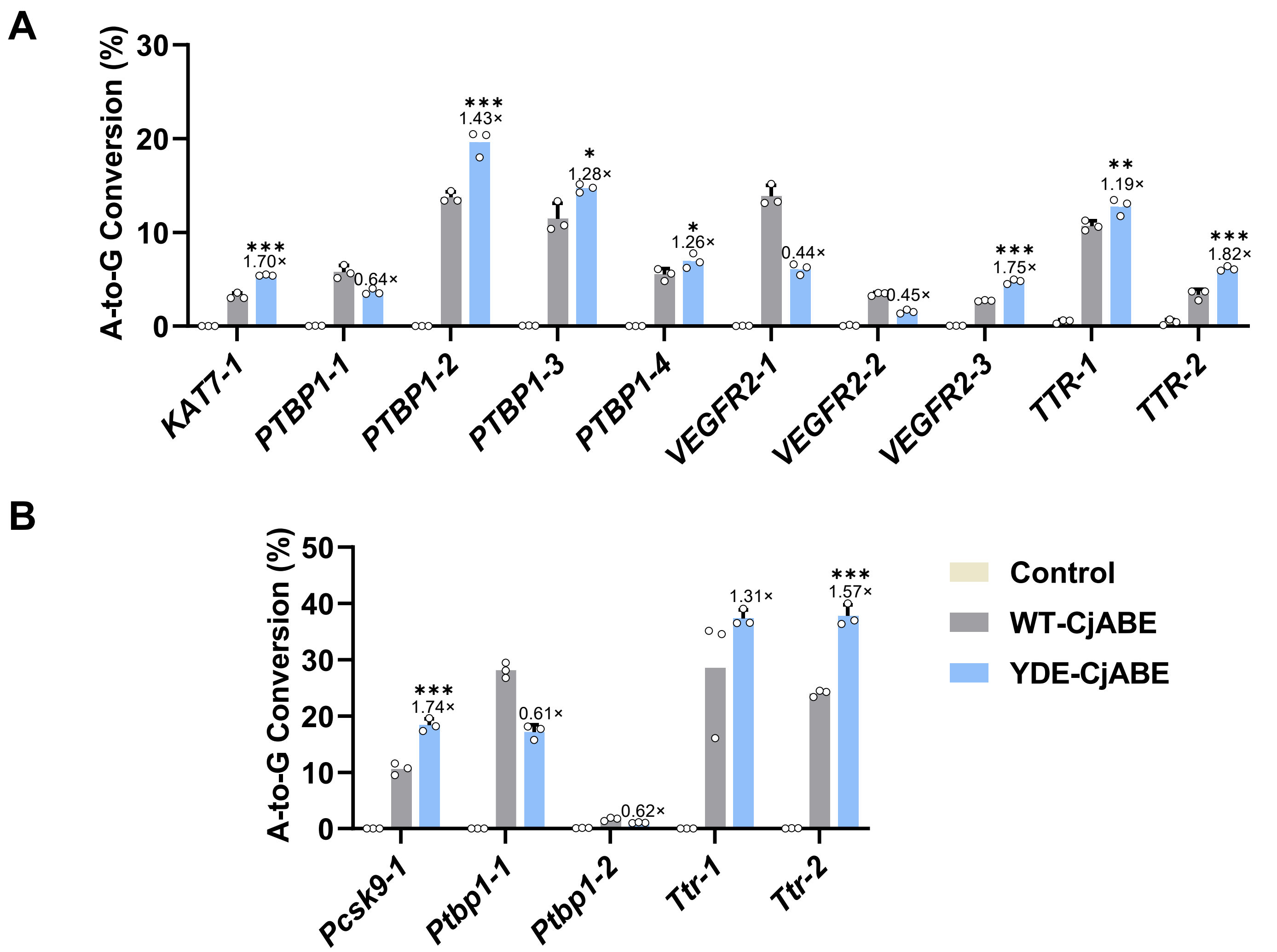

Figure S6. A-to-G editing efficiency of YDE-CjABEs. (A) A-to-G base editing induced by CjABE variants at the endogenous *KAT7, PTBP1, VEGFR2,* and *TTR* target sites in HEK293T cells. (B) A-to-G base editing induced by CjABE variants at the endogenous *Pcsk9, Ptbp1,* and *Ttr* target sites in mouse Hepa1-6 cells. Fold changes comparing to wild type CjCas9 were shown on the top of each bar. Data represented three biological repeats and presented as mean ± S.E.M. Statistical significance was determined using two-tailed t test (*P < 0.05, **P < 0.01, ***P < 0.001)

Figure S7

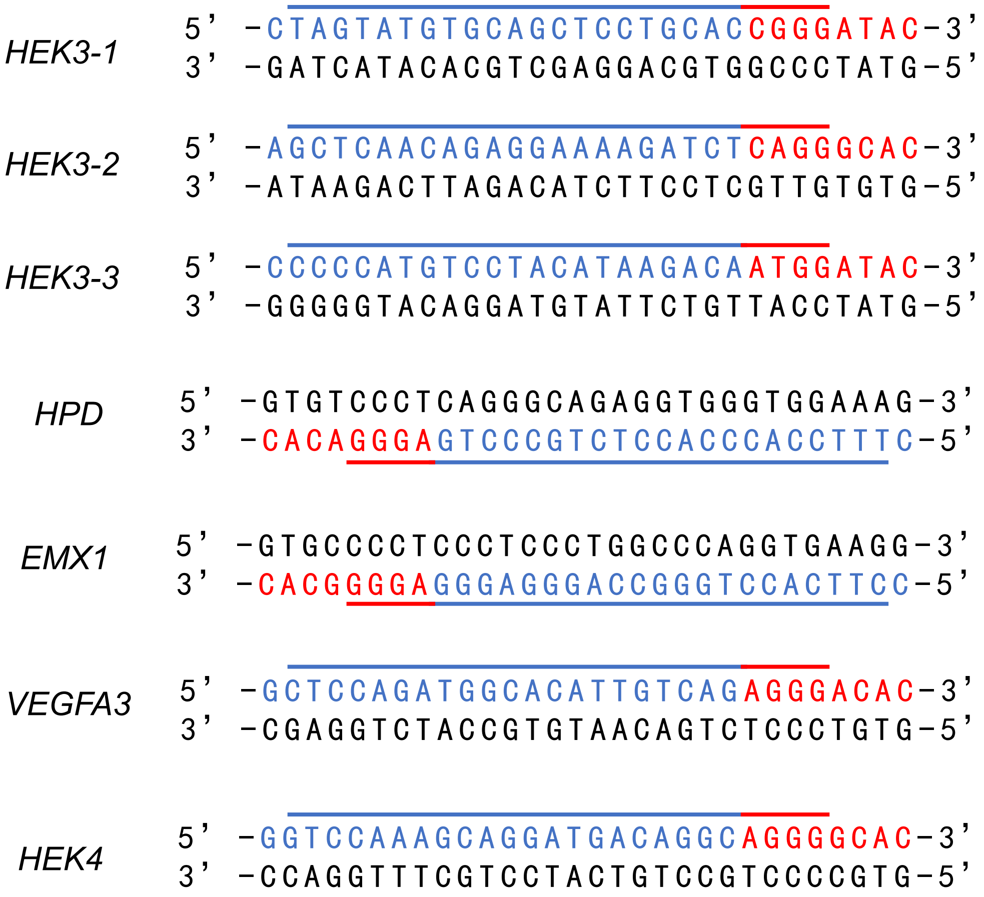

Figure S7. A schematic illustrating the sgRNAs of CjCas9 variant and SauriCas9 targeting for the *HEK3, HEK4, HPD,EMX1,* and *VEGFA3*. The sequence in red represents PAM (Protospacer Adjacent Motif), while the blue represents the spacer referring to Figure 3D. For the CjCas9s, the spacer is directly annotated on the sequence, and for SauriCas9, the spacer is denoted with a horizontal line.

Figure S8

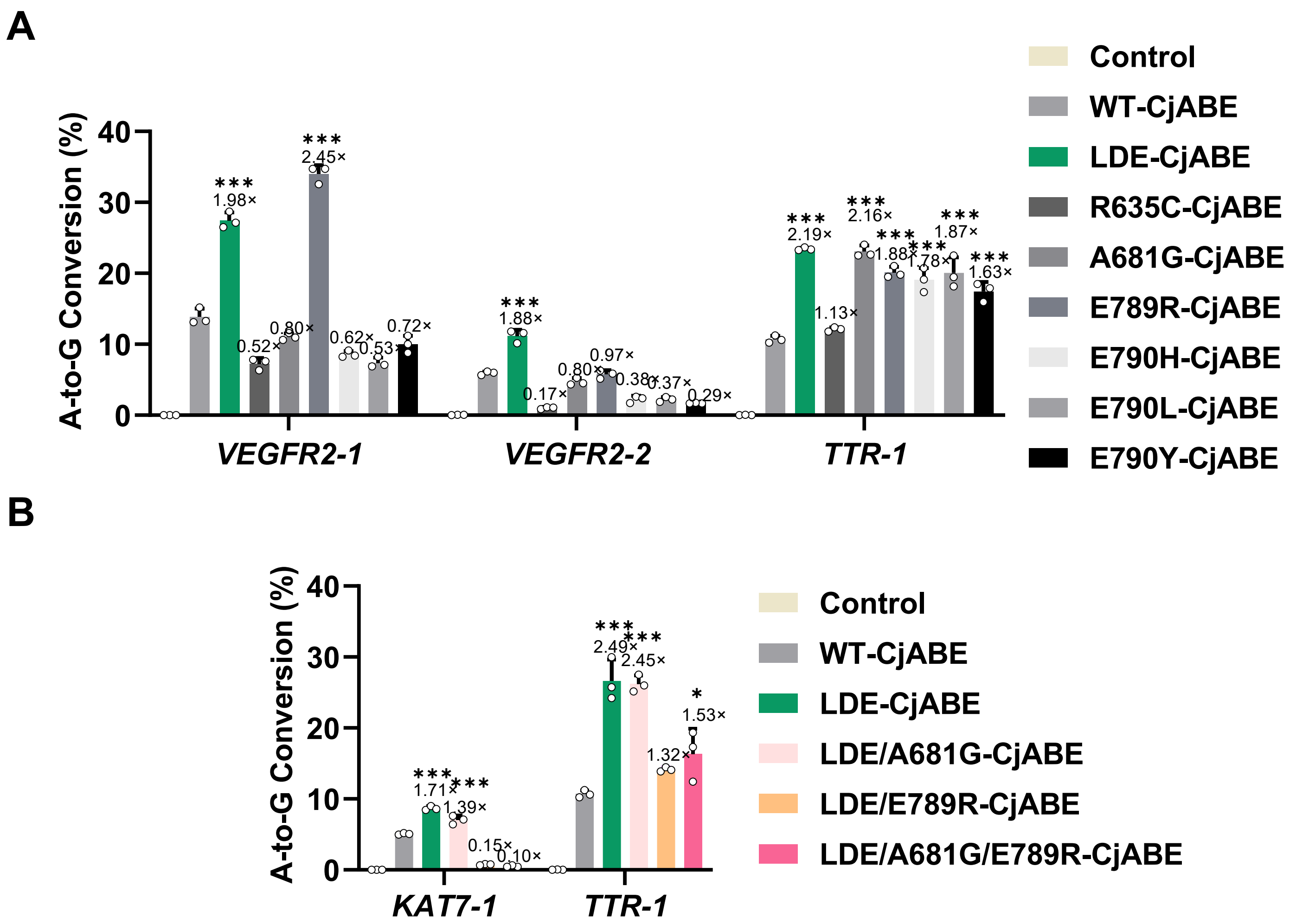

Figure S8. Installing the mutations of AceCas9 to the LDE-CjCas9. (A) A-to-G conversion frequencies of CjABEs at the endogenous *TTR* and *VEGFR2* target sites in HEK293T cells. (B) A-to-G conversion frequencies of CjABEs at the endogenous *KAT7* and *TTR* target sites in HEK293T cells. Control: GFP. Fold changes comparing to wild type CjCas9 were shown on the top of each bar. Data represented three biological repeats and presented as mean ± S.E.M. Statistical significance was determined using one-way ANOVA (*P < 0.05, **P < 0.01, ***P < 0.001)

Figure S9

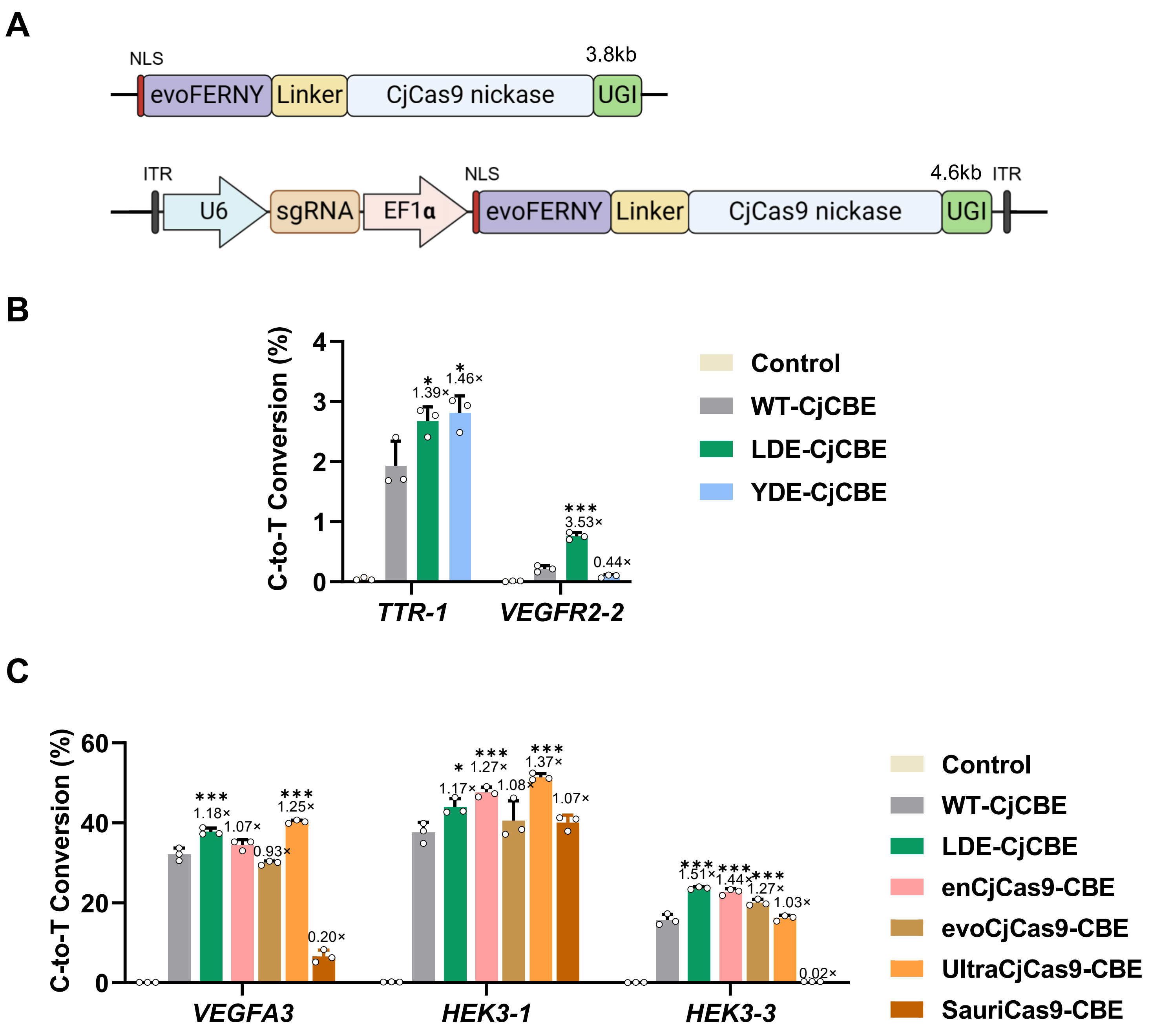

Figure S9. LDE-CjCBE with enhanced gene-editing C-to-T editing efficiency. (A) Schematic overviews of CjCBE constructs. (B) C-to-T conversion frequencies of CjCBEs at the endogenous *TTR* and *VEGFR2* target sites in HEK293T cells. (C) C-to-T conversion frequencies of CjCBE variants and SauriCas9-CBE at the endogenous *VEGFA3* and *HEK3* target sites in HEK293T cells. Control: GFP. Fold changes comparing to wild type CjCas9 were shown on the top of each bar. Data represented three biological repeats and presented as mean ± S.E.M. Statistical significance was determined using one-way ANOVA (*$P < 0.05$, **$P < 0.01$, ***$P < 0.001$)

Figure S10

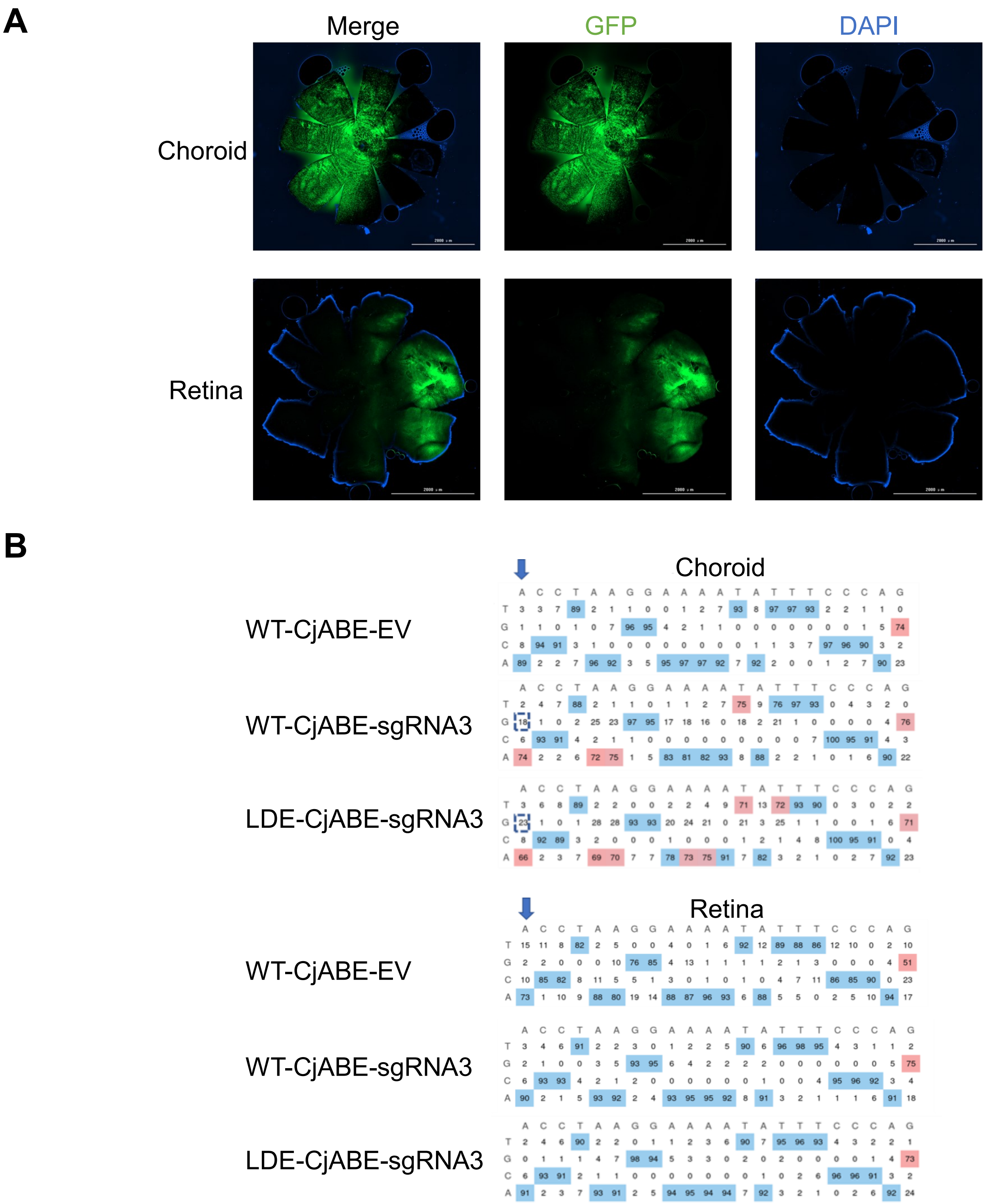

Figure S10. In vivo A-to-G editing efficiency in mouse retina and choroid two weeks post-injection. (A) Representative choroid and retinal flat-mount displaying GFP intensity. Scale bar: 2,000 μm. (B) Targeted A-to-G editing efficiencies of AAV-CjABE at the *Vegfr2-3* in mouse retina and choroid two weeks post-injection. Blue represents the type of base at the site, red indicates the possible base substitutions, and the dashed box represents the target base.

Table S1. CjCas9 amino acids within 3Å of the DNA backbone

Position	Amino Acid	Charge
131	Tyrosine	neutral
145	Isoleucine	neutral
146	Leucine	neutral
191	Tyrosine	neutral
277	asparagine	neutral
392	Aspartic acid	Negatively charged
790	Glutamic acid	Negatively charged
791	Threonine	neutral

Table S2. Comparison of the protein sequences and 3D structure of AceCas9 with CjCas9.

AceCas9	Mutation	AceCas9 3D Structure	CjCas9	Mutation	CjCas9 3D Structure
Arg671	R671C	-	Arg635	R635C	-
Val709	V709G	>4Å	Ala681	A681G	>4Å
Glu839	E839R	3.2-3.9Å	Glu789	E789R	3.2-3.9Å
Glu840	E840H	3.2-3.9Å	Glu790	E790H	3.2-3.9Å
Glu840	E840L	3.2-3.9Å	Glu790	E790L	3.2-3.9Å
Glu840	E840Y	3.2-3.9Å	Glu790	E790Y	3.2-3.9Å

Note: The 3D structural distance represents the distance from an amino acid to DNA.

Table S3. Potential off-target sites predicted by Cas-OFFinder for *Vegfr2_3*.

Off target site	chromosome	position	direction	mismatched base	DNA sequences
On-target	chr5	75944268	+	0	ACCTAAGGAAAATATTTCCCAGAGCAACAC
OT1	chr5	147578322	+	4	ACCTAAGGAgAAgATcTCCCAcAGCAACAC
OT2	chr1	24092659	+	4	ACagAAGtAAAATATTTCCCtGCTAAGCAC
OT3	chr1	130074038	-	4	ACCTAtGGcAAATATTgCCCAtTTTGATAC
OT4	chr12	91657775	+	4	ACCTAgGtAAAccATTTCCCAGAAGAGTAC
OT5	chr17	20342614	+	4	ACCTAAaGAAAATggTgCCCAGGAACATAC
OT6	chr16	50636960	+	4	ACCTAAaGAAAATAgTcCtCAGCAGCACAC
OT7	chr13	36014848	+	4	ACCTAtGGAAgATATTTCtCAtTCAAACAC
OT8	chr18	13287300	-	4	caCTAAGGAAgATcTTTCCCAGGCAGGCAC

Table S4. CjCas9-VPR target sequences.

Target name	Sequence (5' to 3')	PAM
TTN-1	GGAGAGGACCTATTTGGTAGTG	TCACATAC
TTN-2	TCAATCAGAAGAACAGGTGGTA	TGTGACAC
TTN-3	ACTGCCCACAAGTGCTATCTAC	TGTCACAC
HBG	CAGCCGCCGGCCCCTGGCCTCA	CTGGATAC
MIAT	GAAGGCAGGTAGGGACTGGGCT	CAGGGTAC
MPZ	TATAGTCTGGGTGGAGGGGTGG	GGGGACAC
B4GALNT2	ACTATGAGCCAGCCCCTGTGCT	GGGGATAC

Table S5. CjABE and CjCBE target sequences in HEK293T and Hepa 1-6.

Target name	Sequence (5' to 3')	PAM
TTR-1	AACACTCACCGTAGGGCCAGCC	TCGAACAC
TTR-2	CACCGTAGGGCCAGCCTCAGAC	ACAAATAC
Ttr-1	ATCACTCACCGCGGGGCCAGCT	TCAGACAC
Ttr-2	CACCGCGGGGCCAGCTTCAGAC	ACAAATAC
VEGFR2-1	GGAGATGCAACTTACTGCACAA	AGTGACAC
VEGFR2-2	GTTACTCACCTCCTGGCTTGGT	ACAGGCAC
VEGFR2-3	AGGTACCAGACCATGCTGGACT	GCTGGCAC
PTBP1-1	TCGCCGTCTGCAGGACCCACCT	GCCCGCAC
PTBP1-2	CAGCATTGTCCCAGATATAGCC	GTTGGTAC
PTBP1-3	TCCCCAACAGGCCTTCATCGAG	ATGAACAC
PTBP1-4	TTGCTCTGCTGTCTCTAAAGAG	AGTCACAC
Ptbp1-1	CAGCATCGTCCCAGACATAGCA	GTCGGTAC
Ptbp1-2	TTCCTCTGCTGTCTCTAAAGAG	AGTCACAC
KAT7-1	CTAGGAACACAGACAGACCTAT	GGGAACAC
Pcsk9-1	TAGGCCCTGAAGTTGCCCCATG	TGGAGTAC
HEK3-1	CTAGTATGTGCAGCTCCTGCAC	CGGGATAC
HEK3-2	AGCTCAACAGAGGAAAAGATCT	CAGGGCAC
HEK3-3	CCCCCATGTCCTACATAAGACA	ATGGATAC
HEK4	GGTCCAAAGCAGGATGACAGGC	AGGGGCAC
EMX1	CCTTCACCTGGGCCAGGGAGGG	AGGGGCAC
VEGFA3	GCTCCAGATGGCACATTGTCAG	AGGGACAC
HPD	CTTTCCACCCACCTCTGCCCTG	AGGGACAC

Table S6. AAV-CjABE target sequences in Hepa 1-6 and mouse retina.

Target name	Sequence (5' to 3')	PAM
Vegfr2-1	AACGAGGTAACTCACAGCGCAA	AGAGACAC
Vegfr2-2	TATTCTGAATCTGTAGAAGGAG	CAACACAC
Vegfr2-3	ACCTAAGGAAAATATTTCCCAG	AGCAACAC

Table S7. Primer sequences used in this study.

Sequencing primers	Sequence（5’-3’）
DS-H VEGFR2-g1-F	TGATTCCATGTCTCGGGTCC
DS-H VEGFR2-g1-R	ATTCATTGCATTTGACCCTTCCT
DS-H VEGFR2-g2-F	TATTGGTCACCATCTCAATGTGG
DS-H VEGFR2-g2-R	CCAGAGGAGTTGACTGCTTTCC
DS-H VEGFR2-g3-F	AAGTGCTTATTTCAGCATTCAGGA
DS-H VEGFR2-g3-R	ACCTGCTGAGCATTAGCTTGC
DS-H Kat7-g1-F	TGTGGTTTGCATGGATTGAAATG
DS-H Kat7-g1-R	GCTTGTGCTCTTCGGAAAAGAT
DS-H TTR-g1/g2-F	CTTGGCAGGATGGCTTCTCA
DS-H TTR-g1/g2-R	TTGGCAAAGCTGGAAGGAGT
DS-H PTBP1-g1-F	CGTGCAGTCTGTGGTGTCA
DS-H PTBP1-g1-R	TCCTGCCTCACTCCGGG
DS-H PTBP1-g2-F	ACCTACGGGCTCTCCTGG
DS-H PTBP1-g2-R	CAAGGTCAGAGGAGCGTCAC
DS-H PTBP1-g3-F	CCTGAGCCGCGTTTCTCC
DS-H PTBP1-g3-R	AGGGGTCACCGAGGTGTAG
DS-H PTBP1-g4-F	TTCCTCGTGTGGACGATTGG
DS-H PTBP1-g4-R	CTCAGGGCATCAAAATCATCTCA
DS-H HEK4-F	AGGGTCTGGAACCCAGGTAG
DS-H HEK4-R	GAGTCTCCGCTTTAACCCCC
DS-H HEK3-g1-F	GGGTGCCCTGAGATCTTTTCC
DS-H HEK3-g1-R	CTTCCTCCAGAGGGCGTC
DS-H HEK3-g2-F	GGGGCTAGTATGTGCAGCTC
DS-H HEK3-g2-R	CATGCAGGTGCTGAAAGCC
DS-H HEK3-g3-F	ACTTGTTACACGCAGGGCAC

DS-H HEK3-g3-R	CTTTGGGGTTTTCCAGCTGTT
DS-H VEGFA3-F	CCAAAGGACCCCAGTCACTC
DS-H VEGFA3-R	CCAAAGGACCCCAGTCACTC
DS-H EMX1-F	CTCAGTCTTCCCATCAGGCTC
DS-H EMX1-R	GCCCTTCTTCTTCTGCTCGG
DS-H HPD-F	CTTGCCTGGATATGAGGCCC
DS-H HPD-R	CAAGCACCCCTGAGGACTTC
DS-M TTR-g1、2-F	AGAAGCCGTCACACAGATCC
DS-M TTR-g1、2-R	AACTGCCATGTCTGGATCGC
DS-M PTBP1-g1-F	CCTGCCTTTCTAAGCCGGAC
DS-M PTBP1-g1-R	TGACTACAATGGGGTACAATGC
DS-M PTBP1-g2-F	TCTTCCTCGTGGACGATTGG
DS-M PTBP1-g2-R	ACCAATTAAAACGAAACCAAGGTA
DS-M VEGFR2-g1-F	GGCATCGTGTACATCACCGA
DS-M VEGFR2-g1-R	TGAAAGTGGGAAGCTCATCTCC
DS-M VEGFR2-g2-F	GTGGCAATGGGCACACTTAAT
DS-M VEGFR2-g2-R	GAGCAGATACTCACCTCCCG
DS-M VEGFR2-g3-F	GAGCGATGTGTGGTCTTTCG
DS-M VEGFR2-g3-R	ACAGGAGGTAACAGACCGAGA
DS-M PCSK9-g1-F	GAGGTGGACAGTCAGGTGG
DS-M PCSK9-g1-R	TGGGCGAAGACAAAGGAGTC
TTN qFP	TGTTGCCACTGGTGCTAAAG
TTN qRP	ACAGCAGTCTTCTCCGCTTC
GAPDH qFP	GGAGCGAGATCCCTCCAAAAT
GAPDH qRP	GGCTGTTGTCATACTTCTCATGG
MPZ qFP	CTCTCAGGTCACGCTGTATGT
MPZ qRP	GCAGTACCGAACCACGTAGAAA

B4GALNT2 qFP	CCTGCACACGGTTCCCATC
B4GALNT2 qRP	AGCAAGGGTGTTCAGTGTCC
HBG qFP	TGCACTGTGACAAGCTGCAT
HBG qRP	TGGATTGCCAAAACGGTCAC
MIAT qFP	GGTTGGCTCTTTTGTCTTCCAGG
MIAT qRP	GGCTCAGAGAAGTTGCTTGGTC
β-actin-PCR-F	GTCCCTCACCCTCCCAAAAG
β-actin-PCR-R	GCTGCCTCAACACCTCAACCC
AAV-PCR-F	TCCTTCCTGGTGGAAGAGGATAA
AAV-PCR-R	CTCGAACAGCTGGTTGTAGGTC
